# SENP3 loss promotes M2 macrophage polarization and breast cancer progression

**DOI:** 10.1002/1878-0261.12967

**Published:** 2021-05-29

**Authors:** Ming Xiao, Qi Bian, Yimin Lao, Jing Yi, Xueqing Sun, Xuxu Sun, Jie Yang

**Affiliations:** ^1^ Department of Biochemistry and Molecular Cell Biology State Key Laboratory of Oncogenes and Related Genes Shanghai Key Laboratory for Tumor Microenvironment and Inflammation Key Laboratory of Cell Differentiation and Apoptosis of Chinese Ministry of Education Shanghai Jiao Tong University School of Medicine China

**Keywords:** Akt1, breast cancer, macrophage polarization, SENP3, SUMOylation

## Abstract

Tumor‐associated macrophages (TAM) play a crucial role in promoting cancer progression. Upon cytokine stimulation, TAM preferentially polarize to the anti‐inflammatory and pro‐tumor M2 subtype. The mechanism underlying such preferential polarization remains elusive. Here, we report that macrophage‐specific deletion of the SUMO‐specific protease Sentrin/SUMO‐specific protease 3 promotes macrophage polarization towards M2 in bone marrow‐derived macrophage (BMDM) induced by interleukin 4 (IL‐4)/IL‐13 and in an *ex vivo* model (murine Py8119 cell line), as well as in a mouse orthotopic tumor model. Notably, Sentrin/SUMO‐specific protease 3 (SENP3) loss in macrophages accelerated breast cancer malignancy in *ex vivo* and *in vivo* models. Mechanistically, we identified Akt Serine/threonine kinase 1 (Akt1) as the substrate of SENP3 and found that the enhanced Akt1 SUMOylation upon SENP3 loss resulted in Akt1 hyper‐phosphorylation and activation, which facilitates M2 polarization. Analysis of clinical data showed that a lower level of SENP3 in TAM has a strong negative correlation with the level of the M2 marker CD206, as well as with a worse clinical outcome. Thus, increased Akt1 SUMOylation as a result of SENP3 deficiency modulates polarization of macrophages to the M2 subtype within a breast cancer microenvironment, which in turn promotes tumor progression.

## Graphical abstract

Cytokine induced polarization of tumor‐associated macrophages are crucial for cancer progression. Here we investigated the role of SUMO specific peptidase 3 (SENP3) in macrophage polarization. Loss of SENP3 resulted in SUMOylation and hyper‐phosphorylation of Akt1 that in turn facilitated M2 polarization by the cytokines like IL‐4. Altogether, loss of SENP3 contributed to tumor progression and worse prognosis in patients with breast cancer.

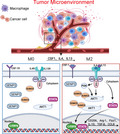

**Abbreviations**
Akt1: Akt serine/threonine kinase 1
Arg‐1: arginase 1
BMDM: bone marrow‐derived macrophage
C/EBPβ: CCAAT/enhancer binding protein (C/EBP), beta
CCL8: C‐C motif chemokine ligand 8
CD206: mannose receptor, C type 1
CD31/Pecam: platelet/endothelial cell adhesion molecule 1
DMEM: Dulbecco's modified Eagle's medium
Drp1: dynamin‐related protein 1
Fizz1: resistin‐like alpha
HE: hematoxylin/eosin
IFN‐γ: interferon γ
IL: interleukin
IRS: insulin receptor substrate
IκB: inhibitor of nuclear factor kappa B kinase
IKBKG: inhibitor of nuclear factor kappa B kinase regulatory subunit gamma
KLF4: Kruppel‐like factor 4
LPS: lipopolysaccharide
M‐CSF: macrophage colony‐stimulating factor
MDSCs: myeloid‐derived suppressor cells
Mgl: metabotropic glutamate receptor
MKK7: MAP kinase kinase 7
MMTV‐PyMT: murine mammary tumor virus‐polyoma middle T antigen
NEMO/IKBKG: inhibitor of nuclear factor kappa B kinase regulatory subunit
PI3K: phosphatidylinositol 3‐kinase
PPARγ: peroxisome proliferator‐activated receptor gamma
SENP3: Sentrin/SUMO specific protease 3
STAT3: signal transducer and activator of transcription 3
STAT6: signal transducer and activator of transcription 6
TAM: tumor‐associated macrophage
TGF‐β: transforming growth factor beta
TLR4: Toll‐like receptor 4
WB: western blot

## 1 Introduction

The tumor microenvironment, which is critical for cancer progression, is composed of multiple types of cells, the soluble substances (e.g. cytokines, hormones, and enzymes) and the extracellular matrix [1, 2, 3, 4]. Among them, the immune cells actively communicate with cancer cells and they influence each other’s function in the tumor niche. Breast cancer is one of the most progressive cancer types in women [5, 6]. Compared with other types of cancer, breast cancer is characterized by having a large population of macrophages, which constitute around 50% of the tumor mass and play a noticeable role in the aggressive behavior of malignant breast cancer [7, 8]. Of note, macrophages are induced to two subtypes, M1 (classically activated macrophages) and M2 (alternatively activated macrophages), referred to as macrophage polarization. M1 macrophages are characterized by the production of pro‐inflammatory cytokines, whereas M2 macrophages are characterized by the production of anti‐inflammatory cytokines [9, 10, 11]. In breast cancer, tumor‐associated macrophages (TAM) are generally characterized as M2 macrophages, which are found to initiate both angiogenesis and invasion to promote tumor progression [12, 13]. Genetically ablating macrophages resulted in a slower rate of tumor progression and a substantial inhibition of metastasis in murine breast cancer models [14, 15]. These studies suggest that TAM appear to be ‘educated’ by cancer cells to polarize towards M2 subtype, which consequently weakens anti‐tumor immune responses and promotes tumor growth and invasion. However, how TAM are preferentially polarized to M2 is largely unknown.

SUMOylation, one of reversible post‐translational modifications (PTM), is involved in various biological processes, such as DNA damage repair, immune responses, and carcinogenesis [16, 17]. SUMO1, SUMO2 or SUMO3 conjugation can be reversed by members of the sentrin/SUMO‐specific protease (SENP) family [18, 19]. This dynamic process is called SUMOylation and de‐SUMOylation. SUMOylation plays an important role in regulating protein stability, localization, activity, and interaction with other proteins, thus altering signal transduction and gene expression in various cells and tissues [20, 21]. It has been reported that Ubc9, a SUMO E2‐conjugating enzyme, inhibits inflammation in Kupffer cells, bone marrow‐derived dendritic cells, macrophages, and RAW264.7 cells [22, 23]. SUMOylation of inflammatory regulators, such as STAT, peroxisome proliferator activated receptor (PPAR) γ, and MKK7, has also been found to inhibit inflammation in macrophages, whereas a pro‐inflammatory role was found for SUMOylation and SENP6‐mediated de‐SUMOylation of NEMO [24, 25, 26, 27]. Our previous study showed that Sentrin/SUMO‐specific protease 3 (SENP3) deficiency in macrophages markedly reduced the production of proinflammatory cytokines, compromised the activation of LPS/Toll‐like receptor 4 (TLR4) signaling, and conferred decreased susceptibility of mice to septic shock [28]. Therefore, SUMOylation and de‐SUMOylation play significant roles in regulating inflammation in macrophages. However, whether and how SUMOylation or SENP3 manipulates M2 polarization is poorly understood, especially during tumor progression.

In this study, we reported that SENP3 deletion in macrophages elicited more M2 polarization and promoted tumor cell proliferation and metastasis within a breast cancer mouse model. We further identified that Akt Serine/threonine kinase 1 (Akt1) phosphorylation and activation facilitated by the increase of its SUMOylation was responsible for enhanced M2 polarization upon SENP3 loss. In human breast cancer tissues, lower SENP3 levels in macrophages were found to be significantly correlated with higher levels of the M2 marker mannose receptor, C type 1 (CD206) and, most importantly, with advanced malignancy, especially lymphatic metastasis. Therefore, our study suggests that macrophages could be educated and polarized to M2 through fine‐tuning Akt1 SUMOylation by SENP3 in response to tumor environment factors. SENP3, as a regulator of macrophage polarization, might serve as a potential therapeutic target and biomarker for lymphatic metastasis of breast cancer.

## 2 Materials and methods

### 2.1 Plasmids

UBC9, RH‐SUMO3, Flag‐SENP3, and Flag‐SENP3 mutant (C532A) plasmids were all constructed in our laboratory [29]. HA‐Akt1 was kindly provided by J. Cheng (Shanghai Jiao Tong University School of Medicine).

### 2.2 Cell culture, transfection, and establishment of stable cell line

HEK‐293T cells were cultured in Dulbecco’s modified Eagle’s medium (DMEM; Gibco, Grand Island, NY, USA) supplemented with 10% fetal calf serum (Biological industries, Kibbutz Beit Haemek, Israel). Transfection with the plasmids was performed using lipofectamine 2000 (Invitrogen, Life Technologies, Carlsbad, CA, USA), according to the manufacturer’s recommendations. Py8119 cells, originally from spontaneously arising tumors in MMTV‐PyMT (mouse mammary tumor virus promoter driven polyoma middle Tantigen) transgenic C57BL/6 female mice, were purchased from ATCC (CRL‐3278) and maintained in F12K nutrient medium (Gibco) and 5% fetal clone II (Fisher Scientific, Pittsburgh, PA, USA), supplemented with MITO Serum Amplifier (BD Biosciences, San Jose, CA, USA). This line is a model for investigating multistep progression of malignant mammary tumorigenesis and metastasis and can also be used as a preclinical mouse model of triple negative breast cancer [30].

Lentiviruses expressing GFP/luciferase dual reporter were produced and provided by Keyuandi Technology Co., Ltd (Shanghai, China). Py8119 cells were infected with a 50 μL of concentrated viral supernatants, along with 10 μg·mL^−1^ polybrene (Sigma Aldrich, St. Louis, MO, USA). Finally, GFP‐positive cells stably expressing luciferase reporter (Luc‐Py8119 for short) were sorted on the FACSAria II flow cytometer (BD Biosciences).

### 2.3 Animal study

C57BL/6 mice were purchased from SLAC Laboratory Animal Co. Ltd (Shanghai, China). Six‐ to 8‐week old mice were used. All mice were maintained under specific pathogen‐free conditions and have free access to food and water. As previously described [28], C57BL/6 Senp3*^fl/fl^* mice were produced by the Model Animal Research Center of Nanjing University (Nanjing, China). *Senp3* conditional KO (cKO) mice were generated by crossing *Senp3^fl/fl^* mice with lysozyme 2 (Lyz2) Cre C57BL/6 mice to delete *Senp3* in macrophages, and then back‐crossed over 20 generations. *Senp3* cKO mice showed no specific phenotype under normal feeding conditions. All animal experiments followed the ‘Guidelines for the Care and Use of Laboratory Animals’ promulgated by the Ministry of Science and Technology of the People’s Republic of China, and were approved by the Institutional Animal Care and Use Committee of Shanghai Jiao Tong University School of Medicine (permit number: A‐2019‐010). Sodium pentobarbital was used to anesthetize the mice to relieve pain.

### 2.4 Human breast cancer tissue microarrays

Three breast cancer tissue microarrays were obtained from Shanghai Outdo Biotech Company (XT14‐047, 28 biopsies, shanghai, China) and Avilabio Biochip Company (BRC01075 and BRC01016, 245 biopsies, Xi'an, China). Each section was prepared according to the standard method, with a diameter of 1.5 mm and a thickness of 4 μm. The experiments were undertaken with the understanding and written consent of each subject. The study methodologies conformed to the standards set by the Declaration of Helsinki. The use of human breast cancer tissue specimens was evaluated and approved by the Ethical Committee of Shanghai Jiao Tong University School of Medicine. The histological diagnosis of each specimen was reconfirmed by microscopic examination of hematoxylin/eosin (HE)‐stained sections.

### 2.5 Breast cancer mouse models

After anesthesia, 2 × 10^5^ Py8119 cells were injected subcutaneously on the left axillary side to form the transplanted breast cancer model. Similarly, 2 × 10^5^ Py8119 cells were injected into the left breast fat pad of mice for orthotopic transplantation. Two or 4 weeks after injection, mice were sacrificed, and tumor tissues, adjacent axillary lymph nodes, and spleen were collected for further examination. Tumors were photographed and weighed. One part of tumors, the adjacent axillary lymph nodes, and spleens were cut for single cell separation for flow cytometry. Another part of the tumors was fixed in 4% paraformaldehyde solution for immunohistochemical staining. The rest of the tumors were frozen in OCT (optimal cutting temperature compound; Sakura, Finetek, Japan) for immunofluorescent staining. The intravenous metastatic breast cancer model was achieved by tail vein injection of 8 × 10^4^ Luc‐Py8119 cells suspended in 200 μL PBS. Two weeks after injection, mice were sacrificed, followed by *in vivo* imaging. Lung tissues were then harvested for HE staining.

### 2.6 Immunohistochemistry

Immunohistochemistry (IHC) was performed in the paraformaldehyde‐fixed and paraffin‐embedded sections of mouse breast tumor tissues and lung tissues. Primary antibodies were used as follows: anti‐mouse‐Ki67 (Abcam, Cambridge, UK, ab15580); anti‐mouse cleaved Caspase 3 (Abcam, ab2302); anti‐mouse CD31 (Maixin, Shanghai, China); anti‐SENP3 (CST, Boston, CA, USA, 5591); anti‐CD206 (Abcam, ab64693). The secondary antibodies (1 : 200) were then incubated for 2 h. Sections were developed with Vectastain ABC kit (CK‐4000) and DAB (SK‐4100) detection system (Vector Laboratories, Burlingame, CA, USA) and counterstained with hematoxylin. The images were photographed, and the average optical density of at least three fields of each image was quantified and statistically analyzed by imagej software (National Institutes of Health, Bethesda, MD, USA).

### 2.7 Bioluminescence imaging and quantification

After 10 min postintraperitoneal injection of luciferin (3 mg/20 g bodyweight, Keyuandi, Shanghai, China), the intensity of *in vivo* biofluorescence of each mouse was evaluated and the pictures were taken using the Lumina II *in vivo* imaging system (Perkin Elmer, Fremont, CA, USA).

### 2.8 Flow cytometry

The samples of well digested tumor suspension were added with 1 μL (0.1 mg·mL^−1^) of fixable viability stain (BD, 564406) in each tube and incubated for 15 min at room temperature. All samples were then blocked with 2 μL Fc blocking solution (0.5 mg·mL^−1^) at 4 °C for 5 min, washed and then incubated with the following specific antibodies on ice for 30 min: anti‐mouse CD16/CD32 (BD, 553141), APC‐Cy™7 anti‐mouse CD45 (BD, 561037), APC anti‐mouse CD11b (BioLegend, San Diego, CA, USA, 101212), PE anti‐mouse F4/80 (BioLegend, 123110), PE‐Cy™7 anti‐mouse CD86 (BD, 560582), PE anti‐mouse CD80 (BD, 561955), Alexa Fluor® 488 anti‐mouse CD206 (BioLegend, 141710), FITC anti‐mouse CD4 (BD, 553046), Alexa Fluor® 647 anti‐mouse CD8a (BioLegend, 100724), PE‐Cy™7 anti‐mouse Ly‐6G and Ly‐6C (BD, 552985). The analysis was performed by Beckman CytoFlex S (Brea, CA, USA).

### 2.9 Sorting of TAM

The 0.5‐cm^3^ breast cancer tissues were dissected and digested in 2.5 mL DMEM (Sigma) with 0.1% collagenase IV (Gibco) at 37 °C on a rotator of 200 r.p.m. for 40 min. Cells were washed and incubated with an FcR blocker at room temperature for 15 min. Cells were then incubated with FITC‐conjugated F4/80 antibody for 15 min, followed by incubation with 100 μL protease cocktail·mL^−1^ sample for 15 min. Finally, FITC‐positive F4/80 TAM were sorted using EasySepTM Mouse FITC Positive Selection Kit II (Stemcell Technologies, Vancouver, Canada).

### 2.10 Quantitative RT‐PCR

Total RNA was isolated from cells and tissues using TRIzol reagent (Invitrogen, Carlsbad, CA, USA). Quantitative RT‐PCR analysis was performed using SYBR Green (Roche, Basel, Switzerland) according to the manufacturer’s instructions on the abi prism® 7500 system (Foster City, CA, USA), as previously described [31]. The primer sequences are listed in Table 1.

**Table 1:** The primers for qRT‐PCR.

Mouse GAPDH	Forward	5 ′ ‐TACAGCAACAGGGTGGTGGAC‐3 ′
	Reverse	5 ′ ‐TGGGATAGGGCCTCTCTTGCT‐3 ′
Mouse SENP3	Forward	5 ′ ‐ACTCCCAGCGAACTCTAA‐3 ′
	Reverse	5 ′ ‐TAATACAAAGGCACCACA‐3 ′
Mouse CD206	Forward	5 ′ ‐CTCTGTTCAGCTATTGGACGC‐3 ′
	Reverse	5 ′ ‐TGGCACTCCCAAACATAATTTGA‐3 ′
Mouse Arg‐1	Forward	5 ′ ‐CTCCAAGCCAAAGTCCTTAGAG‐3 ′
	Reverse	5 ′ ‐GGAGCTGTCATTAGGGACATCA‐3 ′
Mouse IL‐10	Forward	5 ′ ‐CTTACTGACTGGCATGAGGATCA‐3 ′
	Reverse	5 ′ ‐GCAGCTCTAGGAGCATGTGG‐3 ′
Mouse CCL8	Forward	5 ′ ‐CTTCTTTGCCTGCTGCTCATAG‐3 ′
	Reverse	5 ′ ‐CACTTCTGTGTGGGGTCTACA‐3 ′
Mouse C/EBPβ	Forward	5 ′ ‐GAAGACGGTGGACAAGCTGA‐3 ′
	Reverse	5 ′ ‐GCTTGAACAAGTTCCGCAGG‐3 ′
Mouse TGF‐β	Forward	5 ′ ‐CCACCTGCAAGACCATCGAC‐3 ′
	Reverse	5 ′ ‐CTGGCGAGCCTTAGTTTGGAC‐3 ′
Mouse Fizz1	Forward	5 ′ ‐AGTCCCTGCCCTTTGTACACA‐3 ′
	Reverse	5 ′ ‐CGATCCGAGGGCCTCACTA‐3 ′
Mouse Mlg‐1	Forward	5 ′ ‐CAGGATCCAGACAGATACGGA‐3 ′
	Reverse	5 ′ ‐GGAAGCCAAGACTTCACACTG‐3 ′
Mouse VEGF	Forward	5 ′ ‐CTGCCGTCCGATTGAGACC‐3 ′
	Reverse	5 ′ ‐CCCCTCCTTGTACCACTGTC‐3 ′
Mouse MMP2	Forward	5 ′ ‐CAAGTTCCCCGGCGATGTC‐3 ′
	Reverse	5 ′ ‐TTCTGGTCAAGGTCACCTGTC‐3 ′
Mouse MMP9	Forward	5 ′ ‐CTGGACAGCCAGACACTAAAG‐3 ′
	Reverse	5 ′ ‐CTCGCGGCAAGTCTTCAGAG‐3 ′

### 2.11 Bone marrow‐derived macrophage culture

Bone marrow‐derived macrophages were isolated from *Senp3^fl/fl^* or *Senp3^fl/fl,^* Lyz2 Cre mice, as previously described [28]. Briefly, mice were dissected under chloral hydrate anesthesia and sterilized with 75% ethanol. The bones were flushed using a syringe filled with α‐MEM (Gibco) to extrude bone marrow. The cell suspension was filtered and cultured. The next day, the upper layer of mononuclear cells was taken and cultured in α‐MEM supplemented with 10% fetal bovine serum (Gibco). An aliquot of 20 ng·mL^−1^ macrophage colony‐stimulating factor (M‐CSF, Peprotech, Cranbury, NJ, USA) was used to induce cell differentiate. Culture media were changed on the days 3 and 5, and BMDM were matured at day 7.

### 2.12 CCK‐8 analysis

Py8119 cells were planted in a 96‐well plate with a density of 1 × 10^4^ cells per well. A 150‐μL aliquot of conditioned medium from cultured *Senp3* WT BMDM or *Senp3* cKO BMDM was added to Py8119 cells for 0, 12, 24 and 48 h. At the corresponding time point, CCK‐8 reagent was incubated for 2 h, and the absorbance was measured at 450 nm with a microplate reader.

### 2.13 Scratch assay

Py8119 cells were planted in a 6‐well plate with a density of 5 × 10^5^ per well and grown until confluent the next day. The serum was removed and culturing of the cells was continued for 18 h. The cell layer was scraped along a straight line with a pipette tip and the dish gently rinsed with PBS. The scratch image was captured. Py8119 cells were then cultured using the conditioned medium from *Senp3* WT BMDM or *Senp3* cKO BMDM for 24 h, and a wound healing image was photographed again the next day using phase contrast microscope. The area of scratch was analyzed by imagej.

### 2.14 Transwell analysis

Py8119 cells of 5 × 10^4^ were plated in the Transwell chamber of the 24‐well plate, and α‐MEM serum‐free medium, conditioned medium from *Senp3* WT BMDM or *Senp3* cKO BMDM was added to the bottom chamber of the Transwell. The serum‐free medium was added to the upper chamber. After the cells were cultured at 37 °C for 24 h, the upper chamber was fixed in 4% paraformaldehyde for 15 min, and then washed with PBS twice. Finally, the upper chamber was wiped with a cotton swab to remove the cells that did not pass through the Matrigel and the migrated cells were stained with Coomassie Brilliant Blue for 15 min and washed twice with PBS again. After air‐drying, pictures of the chamber were taken under a microscope.

### 2.15 Western blot

The routine method was followed. Briefly, BMDM were harvested to extract proteins and the protein concentration was measured using the BCA method. A 100‐mg protein sample was separated in 8–10% SDS/PAGE and transferred onto poly(vinylidene difluoride) membrane. The primary antibodies were incubated: anti‐SENP3 (CST, 5591), anti‐Akt1 (CST, 2938), anti‐phosphorylated Akt1(Ser473) (CST, 9018), anti‐signal transducer and activator of transcription 6 (STAT6; CST, 5397), anti‐phosphorylated STAT6 (Thr 641) (CST, 56554), anti‐signal transducer and activator of transcription 3 (STAT3; CST, 9139), anti‐phosphorylated STAT3 (Tyr705) (CST, 9145), and anti‐β‐actin (Sigma, AB476744), followed by the secondary antibody incubation (1 : 1000, Sigma). After exposure to the ECL Western Blot (WB) test kit (35050, Thermo, Waltham, MA, USA), visualized images were obtained using ImageQuant TM LAS‐4000 mini (GE Healthcare Life Sciences, Pittsburgh, PA, USA). The protein mass marker was a mixture of 10 to 170 kDa proteins (Thermo Fisher Scientific, 26616).

### 2.16 Immunofluorescence

Frozen sections were permeabilized with 0.2% Triton X‐100, blocked with 10% goat serum, and detected with the following primary antibodies overnight at 4 °C: anti‐SENP3(CST, 5591), anti‐CD206 (Abcam, ab64693), and p‐Akt1(S473) (CST, 9018). After washing, the sections were incubated with a mixture of Alexa Fluor 488‐ and Rhodamine 555‐conjugated secondary antibodies for 1 h at room temperature. Finally, the specimens were washed with PBS and mounted with fluorescent mounting medium containing 4′,6‐diamidino‐2‐phenylindole (DAPI). Cells were then examined under an LSM 880 fluorescence microscope (Zeiss, Oberkochen, Germany).

### 2.17 Akt1 inhibitor treatment

*Senp3* WT and cKO mice were anesthetized with isoflurane, and 2 × 10^5^ Py8119 cells were subcutaneously implanted into the flank area of the mouse. The model mice were injected with p‐Akt1 inhibitor (A‐674563) 20 mg·kg^−1^ bodyweight through the tail vein every other week, and the mice were harvested in the fourth week.

### 2.18 Ni‐NTA pull down assay

Ni‐nitrilotriacetic acid resin (NTA) pull down assay was performed as previously described [31]. Briefly, cells were transfected with RH‐tagged plasmids and then lysed in lysis buffer. The protein samples were harvested and incubated with Ni‐NTA‐Sepharose resin (Qiagen, Dusseldorf, Germany) at 4 °C overnight, according to the manufacturer’s instructions. The resin was then continuously washed with washing buffer at room temperature. After washing, the RH‐tagged proteins were eluted in the elution buffer and subjected to WB.

### 2.19 Co‐immunoprecipitation assay (Co‐IP)

Cells were lysed with IP lysis buffer (50 mm Tris‐HCl, pH 7.4, 150 mm NaCl, 1 mm EDTA, and 1% Triton X‐100) containing protease inhibitor (1 : 100; Promega, Madison, WI, USA), phosphatase inhibitor (1 : 50; Roche), and *N*‐ethylmaleimide (1 : 50; Sigma). The cell lysates were incubated with the corresponding antibody overnight at 4 °C. The next day, protein‐A/G agarose beads (Calbiochem, Temecula, CA, USA) were added and incubated at 4 °C for 4 h. After washing and elution, the samples mixed with loading buffer were examined by WB. Anti‐Flag (Sigma, F3165), anti‐RH (QIAGEN, 34610), and anti‐SUMO2/3 (CST, 4971) antibodies were used.

### 2.20 TIMER

The Tumor Immune Estimation Resource (TIMER) web server is a comprehensive resource for systematic analysis of immune infiltration of various cancer types. The number of six immune cells (B cells, CD4^+^ T cells, CD8^+^ T cells, neutrophils, macrophages, and dendritic cells) in invasive breast cancer was estimated by the TIMER algorithm. We used GSVA to analyze the correlation between the infiltration level of immune cells and the expression level of SENP3 in whole breast cancer tissues.

### 2.21 Kaplan–Meier survival analysis

Kaplan–Meier analysis was used to assess the effect of SENP3 expression on survival of breast cancer patients. SENP3 RNAseq data (Illumina HiSeq, Illumina, San Diego, CA, USA) were obtained from TCGA in invasive breast cancer (updated 10 January 2020). The patients were grouped according to the expression level of SENP3 in the primary tumor tissues: 50% patients with high SENP3 levels and 50% patients with low SENP3 levels.

### 2.22 Statistical analysis

All experiments have been verified by repeated independent experiments. No data were excluded from any analyses and all replicates were true biological replicates. imagej software (Inc, Bethesda, MD, USA) was used to perform densitometric quantification of WB and tumor metastasis‐positive areas. Microsoft excel (San Francisco, CA, USA) or graphpad prism (Graphpad company, La Jolla, CA, USA) was applied to determine the statistical difference. Nonparametric *t*‐test was chosen to compare the differences in clinicopathological features and SENP3 level. Other values were expressed as mean ± SEM, and statistical analyses were performed using one‐way analysis of variance (ANOVA). Spearman rank correlation coefficient was used to analyze the relationship between SENP3 and CD206 expression. The overall survival and recurrence‐free survival rates were estimated using Kaplan–Meier curves, and compared using the log‐rank test. *P* < 0.05 was considered statistically significant.

## 3 Results

### 3.1 SENP3 deletion in macrophages promotes breast cancer progression and metastasis in mouse models

**Fig. 1:**
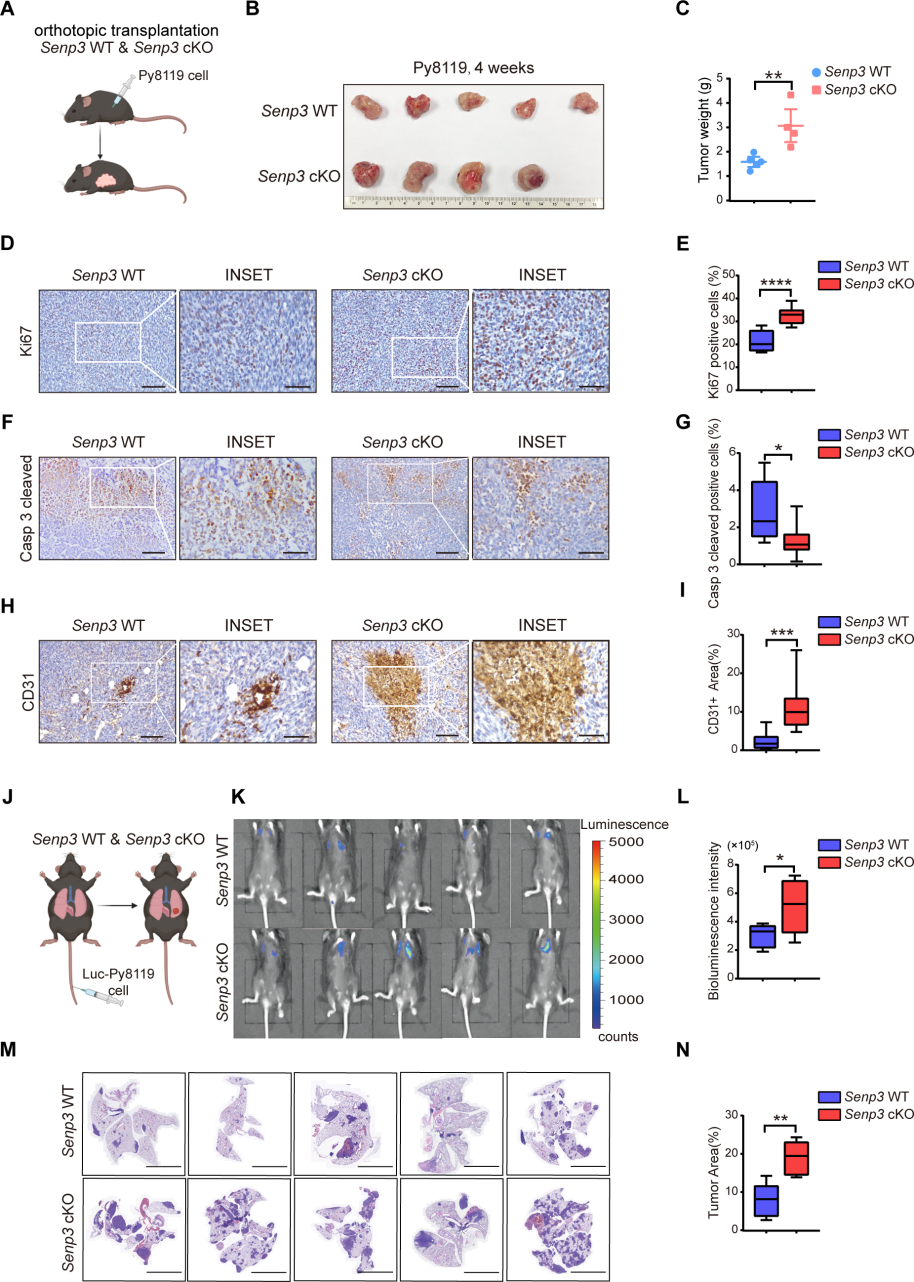
SENP3 deletion in macrophages promotes breast cancer progression and metastasis in mouse models. (A) Schema for breast cancer mouse model. 2 × 10^5^ Py8119 cells were orthotopically or subcutaneously implanted into *Senp3^fl/fl^* (referred *Senp3* WT) and *Senp3* cKO mice. (B) Size of orthotopic tumor. Scale bar: 1 cm. (C) The weight of orthotopic tumor (*n* = 5 in Senp3 WT, *n* = 4 in Senp3 cKO). (D,E) Ki67 staining of transplanted tumors. (F,G) Cleaved caspase 3 staining of transplanted tumors. (H,I) CD31 staining of transplanted tumors. Representative images are shown. Scale bars: 50 (D,F) and 20 μm (H). Graphs were shown as mean ± SD in (E,G,I) (*n* = 5 in *Senp3* WT, *n* = 6 in *Senp3* cKO). (J) Schema for metastasis breast cancer model. 8 × 10^4^ Py8119 cells with luciferase reporter (Luc‐Py8119) were injected into tail vein. (K,L) Bioluminescence imaging and quantification of bioluminescence intensity at 2 weeks postinjection. (M,N) Lung metastasis from *Senp3* WT and *Senp3* cKO mice. Scale bar: 1 cm. Graphs are shown as mean ± SD in (L, N). (*n* = 5 in *Senp3* WT and in *Senp3* cKO respectively). Two‐tailed unpaired Student’s *t*‐tests were performed. *P < 0.05, **P < 0.01, ***P < 0.001, ****P < 0.0001. The experiments were repeated two (metastasis model) or three times (subcutaneous inoculation model) and representative results are shown.

To determine whether SENP3 plays a role in macrophages during breast cancer progression, we first generated macrophage‐specific *Senp3* knockout mice (*Senp3* cKO) by crossing *Senp3^fl/fl^* mice with lysozyme 2 (Lyz2) Cre mice; the disruption of *Senp3* in BMDM was then verified by quantitative RT‐PCR (qRT‐PCR) analysis (Fig. S1A). Next, Py8119 breast cancer cells were orthotopically injected into *Senp3^fl/fl^* mice (referred to as *Senp3* WT hereafter) and *Senp3* cKO mice, respectively (Fig. 1A). We found that the size and weight of tumors were significantly higher in the *Senp3* cKO group than in *Senp3* WT mice 4 weeks postimplantation (*P* < 0.01; Fig. 1B,C). Moreover, in a subcutaneous injection with Py8119 cells, besides higher tumor weight, we observed more spontaneous lung metastatic sites in *Senp3* cKO mice 4 weeks after transplantation (Fig. S1B–E). Consistently, increased proliferation and decreased apoptosis were observed in post‐4‐week tumor tissues from *Senp3* cKO mice, as evidenced by immunohistochemical (IHC) staining of Ki67 and cleaved caspase‐3, respectively (Fig. 1D–G). In addition, platelet/endothelial cell adhesion molecule 1 (Pecam)/CD31 staining indicated enhanced tumor angiogenesis in tumors from *Senp3* cKO mice (Fig. 1H,I). These results demonstrate that *Senp3* deletion in macrophages promotes breast cancer progression.

To further explore whether SENP3 loss in macrophages is associated with cancer metastasis, we established a metastatic mouse model by injecting Luc‐Py8119 cells through the tail vein (Fig. 1J). The bioluminescence and histologic analyses showed increased lung colonization of the injected breast cancer cells in *Senp3* cKO mice compared with WT mice (Fig. 1K–N). These data indicated that SENP3 deficiency in macrophages promotes tumor growth and metastasis. Given that TAM is well‐known to promote cancer progression, these results also suggest that *Senp3* might regulate TAM function in tumor niches and further affect the crosstalk between TAM and cancer cells.

### 3.2 Loss of SENP3 facilitates macrophage polarization towards M2 within breast cancer microenvironment

**Fig. 2:**
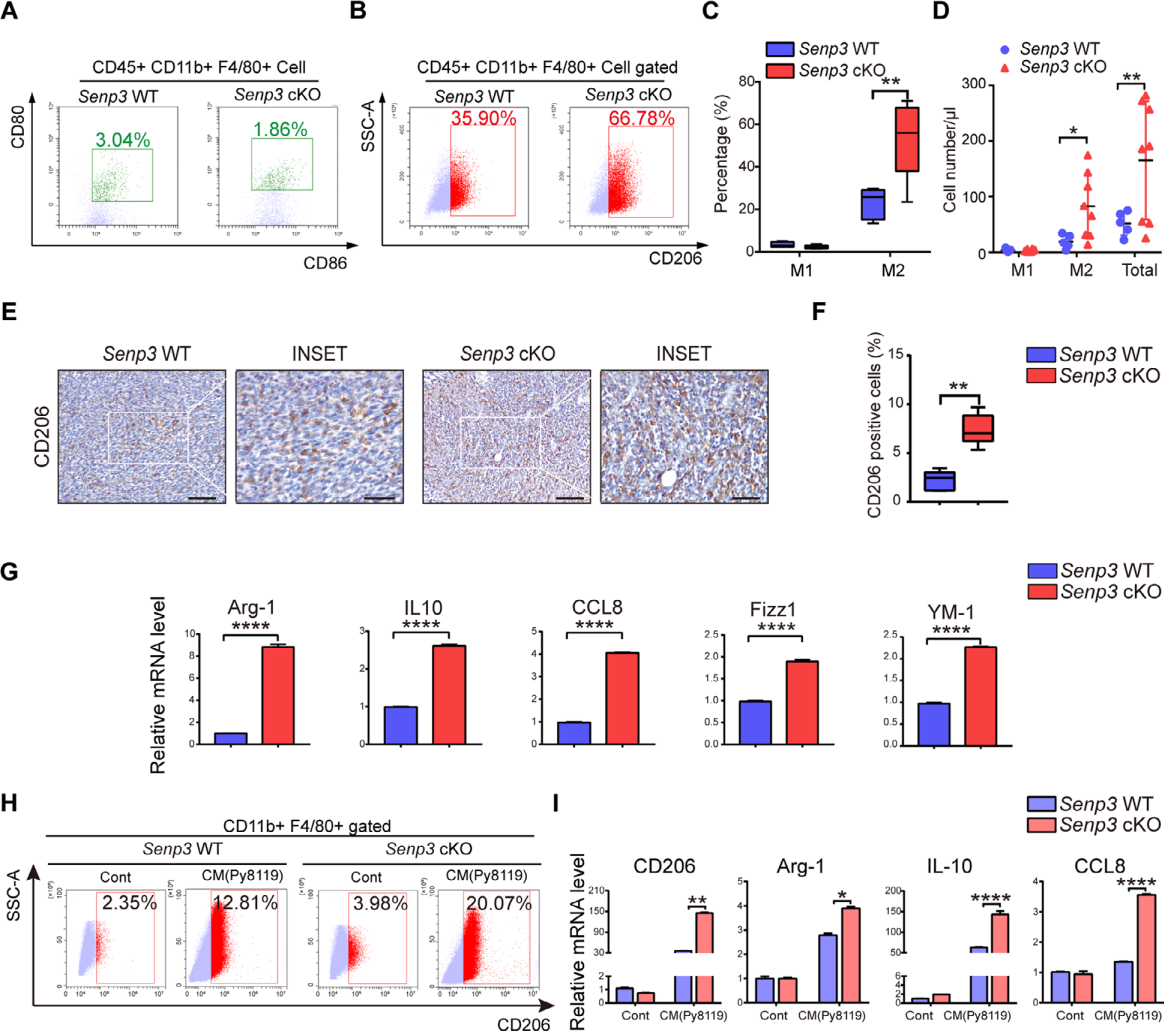
Loss of SENP3 facilitates macrophage polarization towards M2 within breast cancer microenvironment. (A–D) Isolated cells from transplanted breast cancer tissues were analyzed and gated with BMDM markers CD45^+^ CD11b^+^ F4/80^+^ by flow cytometry. (A) M1 macrophages with CD80^+^/CD86^+^. (B) M2 macrophages with CD206^+^. (C) Quantification is shown as mean ± SD (*n* = 5 in *Senp3* WT, *n* = 6 in *Senp3* cKO). (D) The cell numbers of M1, M2 and total macrophages, calculated according to flow cytometry data. Tumor tissues with the same weight were resolved into the same volume of lysis buffer, the cell concentration was calculated according to the cell number and the volume of buffer detected by flow cytometry. Quantification is shown as mean ± SD (*n* = 5 in *Senp3* WT, *n* = 6 in *Senp3* cKO). (E) CD206 in transplanted tumors. The representative pictures are shown. Scale bars: 50 and 20 μm. (F) Quantification is shown as mean ± SD (*n* = 5 in *Senp3* WT, *n* = 6 in *Senp3* cKO). (G) The mRNA levels of Arg‐1, IL‐10, CCL8, Fizz1, and YM‐1 in macrophages within tumor tissues. *Senp3* WT and *Senp3* cKO macrophages were sorted by flow cytometry and the transcription of indicated genes was monitored by qRT‐PCR. Quantification is shown as mean ± SD (*n* = 5 in *Senp3* WT, *n* = 6 in *Senp3* cKO). The experiments in mouse breast cancer model were reproduced three times and representative results are shown. (H) M2 macrophages with CD206^+^. *Senp3* WT and *Senp3* cKO BMDM were induced by Py8119 cell culture medium [conditioned medium (CM)]. CD206 was analyzed by flow cytometry 48h postinduction. (I) The mRNA levels of CD206, Arg‐1, IL‐10, and CCL8 in BMDM. *Senp3* WT and *Senp3* cKO BMDM were induced by CM of Py8119 for 6 h. The experiments were repeated three times and quantification is shown as mean ± SD. Two‐tailed unpaired Student’s *t*‐tests were applied. *P < 0.05, **P < 0.01, ****P < 0.0001. The scale bar of the inset picture is 20 μm, and the scale bar of the original picture is 50μm.

To explore the effect of SENP3 loss on TAM function in tumor niches, we isolated TAM from the Py8119 transplants in *Senp3* WT and cKO mice and analyzed the percentages of M1 and M2 macrophages within the TAM population. The result of flow cytometry showed that the percentage of M1 macrophages (CD80^+^ CD86^+^) was low in both *Senp3* WT and cKO groups, and there was no statistical difference between them (Fig. 2A). However, the percentage of M2 macrophages (CD206^+^) in transplants from *Senp3* cKO mice exhibited about a twofold increase compared with that from WT mice (Fig. 2B–D). Consistently, IHC revealed more CD206^+^ cells in the tumor tissues from *Senp3* cKO mice (Fig. 2E,F). The expression of other M2 marker genes, including *Arg‐1, IL‐10, CCL8, Fizz1 and YM‐1*, were also upregulated in the tumor from *Senp3* cKO mice, as demonstrated by qRT‐PCR analysis (Fig. 2G). We further examined whether other immune cells were affected by SENP3 deficiency in macrophages, including myeloid‐derived suppressor cells (MDSCs), CD4^+^ and CD8^+^ T cells isolated from tumor tissues, spleen, and tumor‐draining lymph nodes. None of these cell populations showed any difference between *Senp3* WT and cKO mice (Fig. S2A–F). Thus, these results indicated that loss of SENP3 in macrophages facilitates TAM polarization into the M2 subtype in breast cancer transplants without affecting other types of immune cells.

To determine further whether the effect of SENP3 loss on macrophage polarization requires the presence of tumor cells, we generated primary BMDM from WT and *Senp3* cKO mice and cultured them in conditioned media (CM) from Py8119 cells. Although M2 polarization in BMDM was barely induced in the absence of CM, regardless of the expression of SENP3, culturing with the conditional media for 24 h obviously induced substantial amounts of BMDM into M2 in both groups (Fig. 2H,I). More importantly, 20.07% BMDM from *Senp3* cKO mice was differentiated to M2, whereas only 12.81% BMDM from WT mice adopted the M2 phenotype (Fig. 2H). Consistently, the expression levels of M2 marker genes, including CD206, arginase‐1 (Arg‐1), C–C motif chemokine ligand‐8 (CCL‐8), and IL‐10, were significantly higher in BMDM from *Senp3* cKO mice than counterparts from WT mice (Fig. 2I). These data indicate that macrophages with SENP3 deficiency preferentially polarize to the M2 subtype in response to tumor cell‐secreted factors.

As another mouse model, melanoma cells B16F10 were subcutaneously injected in mice. Similarly, the transplants from *Senp3* cKO were bigger and contained more CD206^+^ cells (Fig. S3A–E). SENP3 deficiency in macrophages did not result in any fluctuation of the ratio of CD86^+^ cells (Fig. S3D,E). Collectively, SENP3 appears to act as a specific regulator for M2 polarization for various types of tumor.

### 3.3 SENP3 deletion in macrophages promotes M2 polarization, resulting in increased proliferation and migration of breast cancer cell

**Fig. 3:**
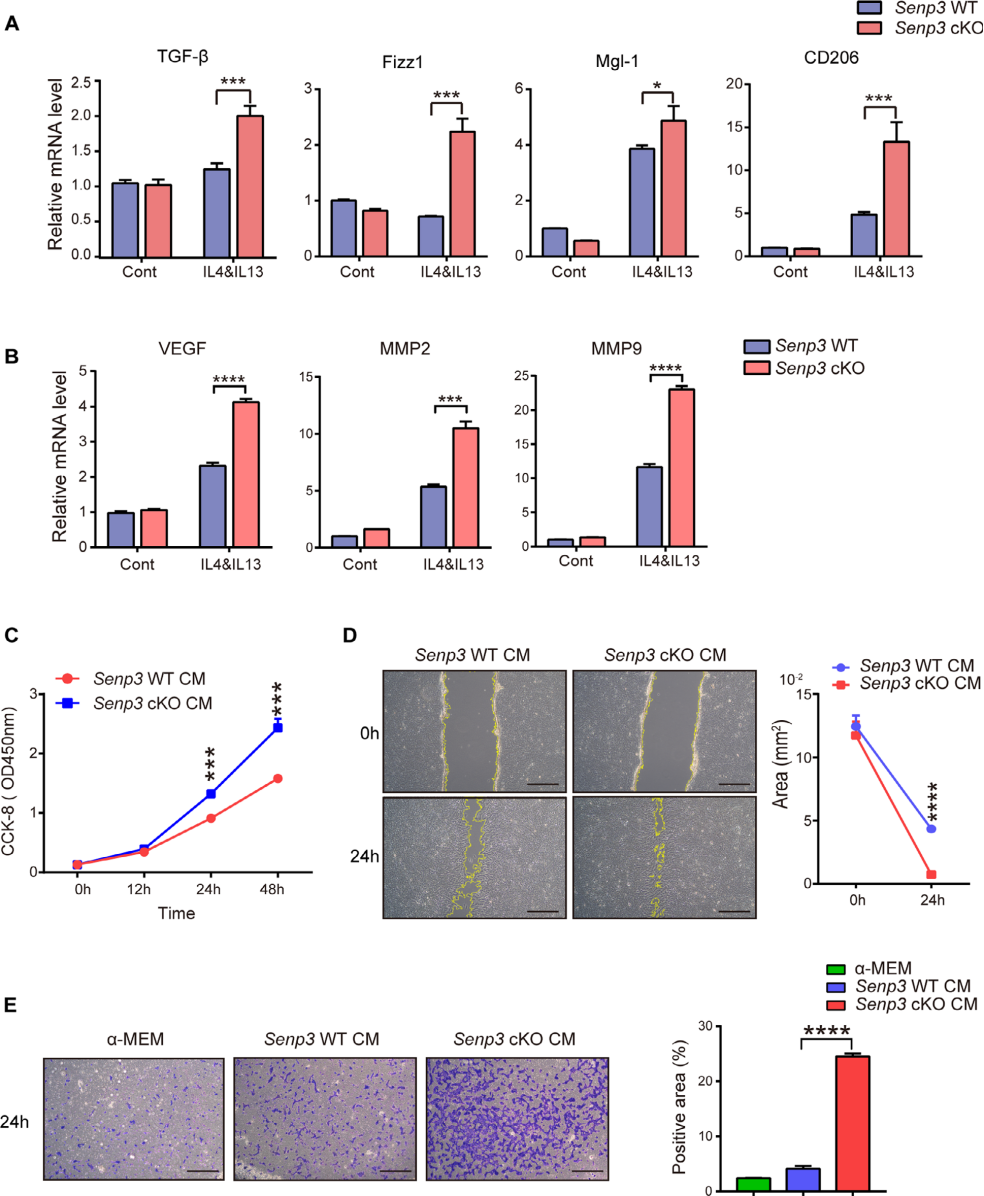
SENP3 deletion in macrophages promotes M2 polarization resulting in increased proliferation and migration of breast cancer cells. BMDM were treated with IL‐4 and IL‐13 (20 ng·mL^−1^) for 6 h. (A) The mRNA levels of TGF‐β, Fizz1, Mgl‐1, and CD206. (B) The mRNA levels of VEGF, MPP2 and MPP9. (C–E) The proliferation, migration, and invasion of Py8119 induced by the conditioned medium of *Senp3* WT or *Senp3* cKO macrophages. CCK‐8 assay, scratch experiment, and Transwell assay were performed, respectively. The experiments were repeated three times and quantification is shown as mean ± SD. Representative images are shown. Scale bar: 200 μm. Two‐tailed unpaired Student’s *t*‐tests were applied. *P < 0.05, ***P < 0.001, ****P < 0.0001.

*In vitro* IL‐4 and IL‐13 were applied in combination to BMDM to induce M2 macrophage. In line with CD206 upregulation in the tumor tissues within cKO mice, other M2 subtype markers, such as transforming growth factor (TGF‐β), resistin‐like molecule alpha/Fizz1, metabotropic glutamate receptor (Mgl‐1), vascular endothelial growth factor (VEGF), matrix metallopeptidase 2 (MMP2) and MMP9, were notably increased in *Senp3* cKO BMDM 6 h after treatment with IL‐4/IL‐13 (Fig. 3A,B). CM from *Senp3* cKO BMDM induced by IL‐4/IL‐13 also notably accelerated proliferation, migration, and invasion of Py8119 cells (Fig. 3C–E). These results suggest that in an *in vitro* model consisting two types of cells, macrophage and breast cancer cells, SENP3 might promote tumor progression by modulating macrophage polarization.

### 3.4 SENP3 loss promotes M2 polarization of macrophage through activating Akt1 *in vitro*

**Fig. 4:**
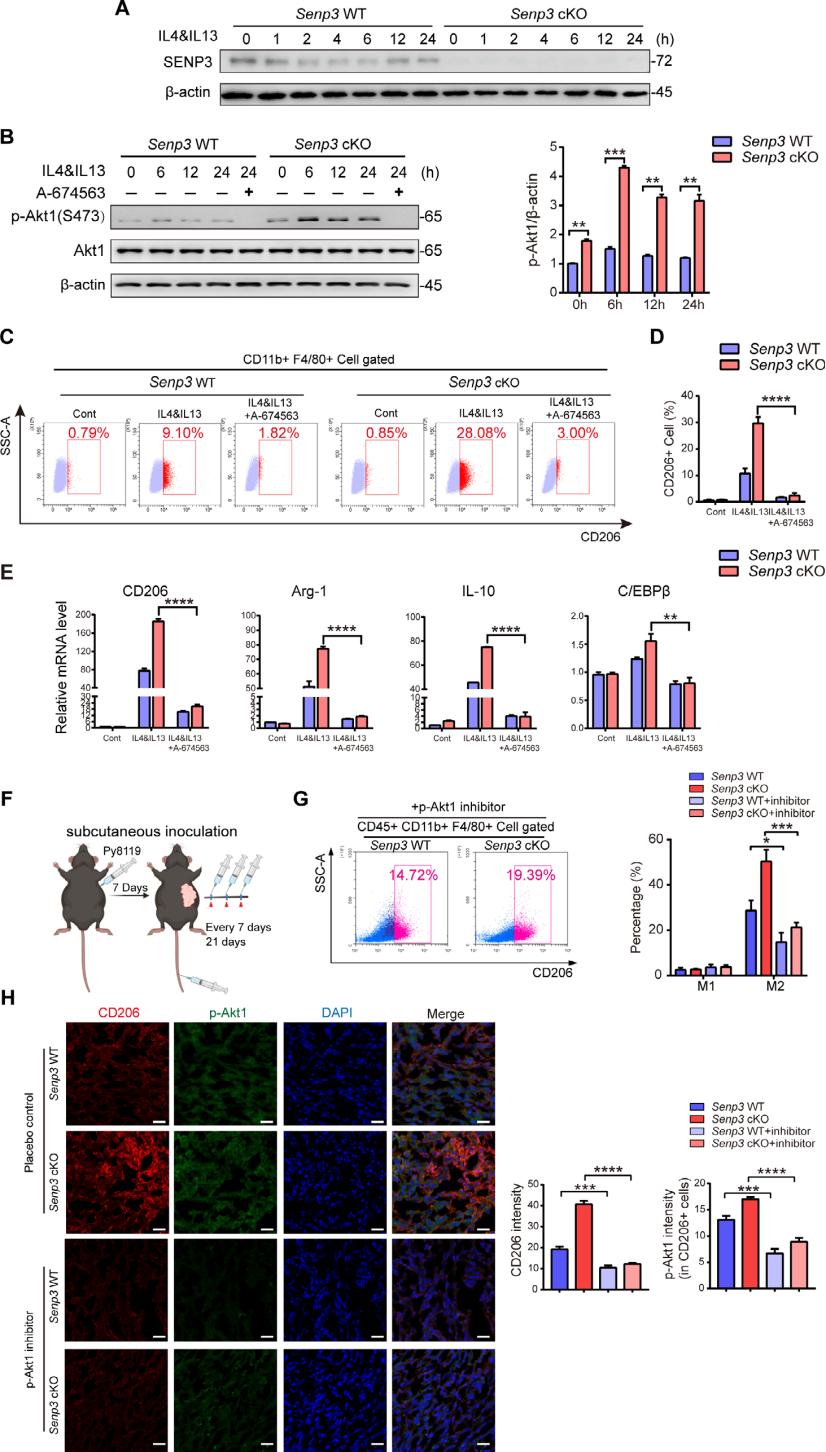
Loss of SENP3 promotes M2 polarization of macrophage through activation of Akt1. (A,B) *Senp3* WT and *Senp3* cKO BMDM were treated with IL‐4 and IL‐13 for the times indicated. (A) SENP3 level. (B) The phosphorylated Akt1 at S473 (p‐Akt1 for short). The experiments were repeated three times and representative images are shown. The quantification of the levels of p‐Akt1 is shown as mean ± SD. (C–E) *Senp3* WT and *Senp3* cKO BMDM were treated with IL‐4 and IL‐13 (20 ng·mL^−1^) for 24 h in the presence or absence of A‐674563 (5 mm). A‐674563 was pretreated for 12 h when used. (C,D) M2 macrophages with CD206^+^. The experiments were repeated three times. The representative results are shown in (C) and quantification is shown as mean ± SD in (D). (E) The mRNA levels of CD206, Arg‐1, IL‐10, and C/EBPβ. Quantification is shown as mean ± SD of three independent experiments. (F) Diagram for p‐Akt1 inhibitor administration in breast cancer mouse model. Py8119 cells 2 × 10^5^ were subcutaneously transplanted in *Senp3* WT and *Senp3* cKO mice. A 20 mg·kg^−1^ aliquot of p‐Akt1 inhibitor was injected every week after modeling, and tumors harvested at the fourth week. (G) M1 macrophages with CD80^+^, CD86^+^, and M2 macrophages with CD206^+^. Isolated cells from transplanted cancer tissues were analyzed and gated with CD45^+^ CD11b^+^ F4/80^+^ by flow cytometry. Quantification is shown as mean ± SD of three mice in every group. (H) The intensity of CD206 and *p*‐Akt1 in macrophages within transplanted tumor tissues. Co‐immunofluorescence was performed with indicated antibodies. Representative images are shown. Scale bar: 20 μm. Quantification is shown with the mean ± SD of three mice in every group (*n* = 3, ***P < 0.001, ****P < 0.0001). Two‐tailed unpaired Student’s *t*‐tests were performed for statistical analysis. *P < 0.05, **P < 0.01, ***P < 0.001, ****P < 0.0001 in (B,D,E, and G), respectively.

Upon IL‐4/IL‐13 treatment, SENP3 protein levels declined at a very early time point (Fig. 4A), and SENP3 mRNA levels were downregulated as well at 6 h (Fig. S4), suggesting that the reduction of SENP3 may be caused by decreasing its transcription and possibly accelerating its degradation in response to cytokine stimulation. We then examined major pathways involved in M2 polarization upon *Senp3* loss. We found that *Senp3* loss had no effects on the phosphorylation of STAT3 at Y705 and STAT6 at T641 upon IL‐4/IL‐13 treatment in BMDM (Fig. S5A,B). However, the phosphorylation of Akt1 at S473 (p‐Akt1) was intensely increased in *Senp3* cKO BMDM (Fig. 4B), suggesting that SENP3 deficiency activates the Akt pathway, rather than the STAT6 or STAT3 pathway. Given that SENP1, another member of SENP family, was reported to regulate Akt activation [32], we further compared the role of both SENP on Akt activation in BMDM. We found that the phosphorylation levels of Akt1 were slightly reduced upon *Senp1* knockout, compared with those in WT group (Fig. S5C), indicating a specific effect of SENP3 on Akt inhibition.

Next, we determined whether *Akt1* hyper‐activation indeed promotes M2 polarization in *Senp3*‐deficient macrophages. We found that inhibition of *Akt1* by A674563, a selective Akt1 inhibitor, completely blocked M2 polarization in both *Senp3* WT and cKO BMDM (Fig. 4C,D). Consistently, the induction of the expression of several M2 marker genes, including *CD206*, *Arg‐1* and *IL‐10*, as well as the M2‐related transcription factor CCAAT/enhancer binding protein β (*C/EBPβ*), was also abrogated upon A674563 preincubation (Fig. 4E). Moreover, A674563 treatment significantly decreased the percentage of CD206^+^ macrophages in tumor tissues to a similar extent in cKO mice and WT mice bearing Py8119 transplants (Fig. 4F,G). The increase in the intensity of *CD206* and *p‐Akt1* in CD206^+^ cells in *Senp3* cKO mice was abrogated by A674563 as well (Fig. 4H). Although tumor growth was significantly reduced after systematic treatment of the inhibitor (Fig. S6A,B), inhibition of Akt1 in M2 macrophage contributed to suppression of tumor growth, which was accelerated by SENP3 loss in macrophages. These results indicate that SENP3 may inhibit Akt1 activation to suppress M2 polarization, leading to the suppression of breast cancer progression.

### 3.5 Loss of SENP3 in macrophages enhances Akt1 SUMOylation to induce its hyperphosphorylation

**Fig. 5:**
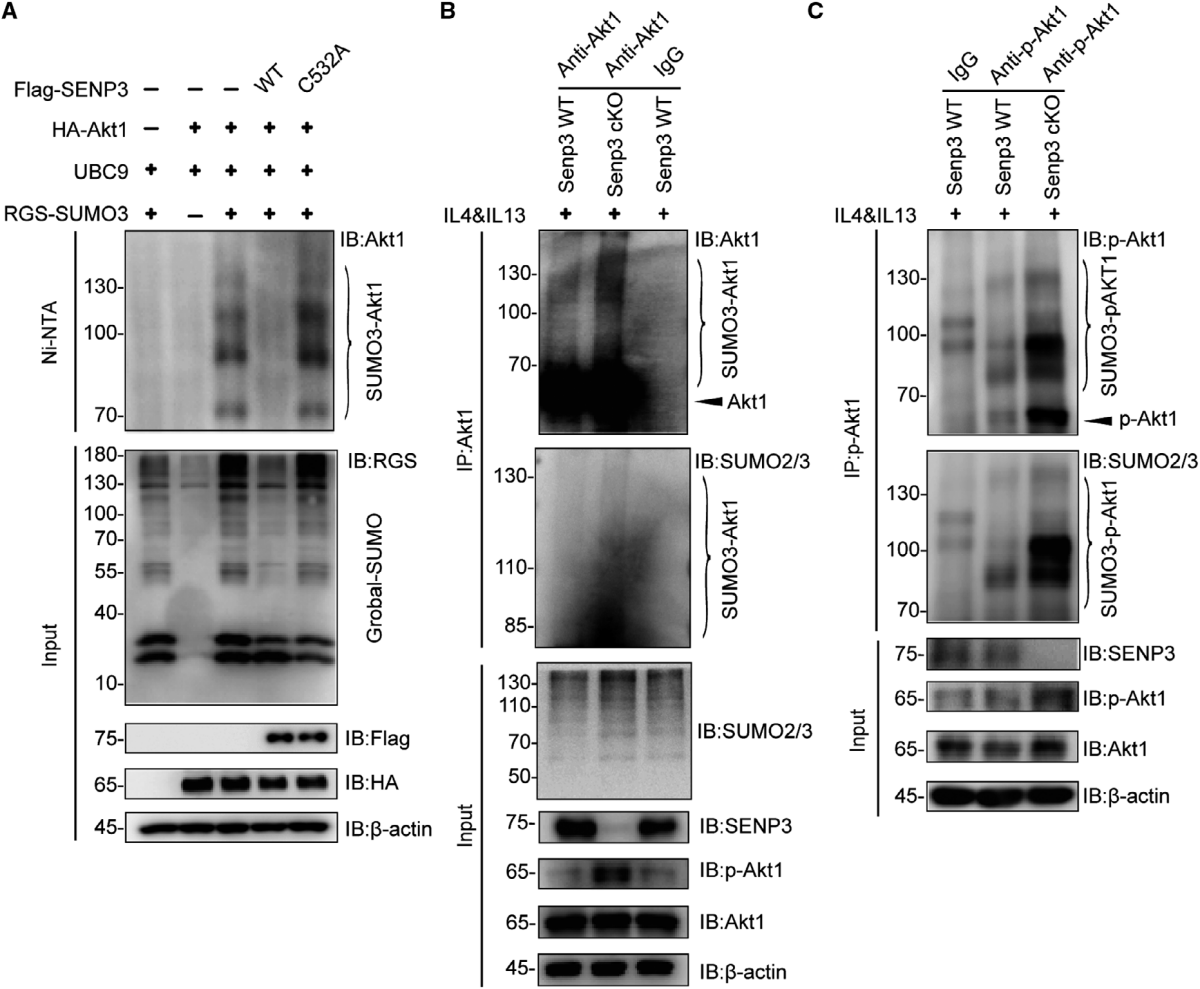
Loss of SENP3 in macrophage enhances Akt1 SUMOylation to promote its hyperphosphorylation. (A) SUMO3 conjugation on Akt1 was determined by Ni‐NTA pull down. HA‐Akt1, RH‐SUMO3, and UBC9 were co‐transfected into HEK293T cells along with Flag‐SENP3 or Flag‐SENP3 C532A mutant for 48 h. RH‐SUMO3 was pulled down using Ni‐NTA resin and analyzed by WB with indicated antibodies. (B) The endogenous SUMOylation of Akt1 in BMDM was detected by co‐IP. BMDM were treated with IL‐4 and IL‐13 (20 ng·mL^−1^) for 12 h. (C) The endogenous SUMOylation of p‐Akt1 (S473) in BMDM was detected by co‐IP. BMDM were treated with IL‐4 and IL‐13 (20 ng·mL^−1^) for 12 h. The experiments were repeated three times.

To elucidate further the mechanisms underlying the increase in Akt1 phosphorylation caused by SENP3 deficiency, we next investigated whether SENP3 regulates Akt1 de‐SUMOylation by affecting its phosphorylation, given that Akt1 is known to be SUMOylated [33]. We first overexpressed HA‐Akt1 with SUMO2/3 and SENP3 WT or catalytic mutant (SENP3 C532A) in HEK293T cells. The result showed that Akt1 was indeed modified by SUMO2/3, consistent with the previous report [33]. Notably, SENP3 WT, but not the C532A mutant, remarkably reduced SUMO3 conjugation on Akt1, indicating that Akt1 is a substrate of SENP3 (Fig. 5A). We then detected Akt1 SUMOylation in primary BMDM from *Senp3* WT and cKO mice upon treatment with IL‐4 and IL‐13. The result showed that SUMO2/3 conjugating to endogenous Akt1 was obviously increased in BMDM from *Senp3* cKO mice (Fig. 5B). To examine the interplay between Akt1 SUMOylation and phosphorylation, we performed pull down assay and found that p‐Akt1 was also conjugated with SUMO2/3, and SENP3 loss dramatically increased SUMO modification of p‐Akt1 (Fig. 5C). By observing and comparing the band intensity, we found higher levels of SUMOylation of p‐Akt1 than of total Akt1 in *Senp3*‐deficient BMDM (Fig. 5B,C), suggesting that SUMOylation might facilitate phosphorylation of Akt1. Moreover, we found that SENP3 was localized in the cytosol of CD206^+^ cells, (Fig. S7), which was similar to Akt. Altogether, these data indicate that deleting SENP3 enhances Akt1 SUMO2/3 modification, which promotes Akt1 activation in BMDM.

### 3.6 SENP3 level is negatively correlated with M2 polarization in breast cancer patients

**Fig. 6:**
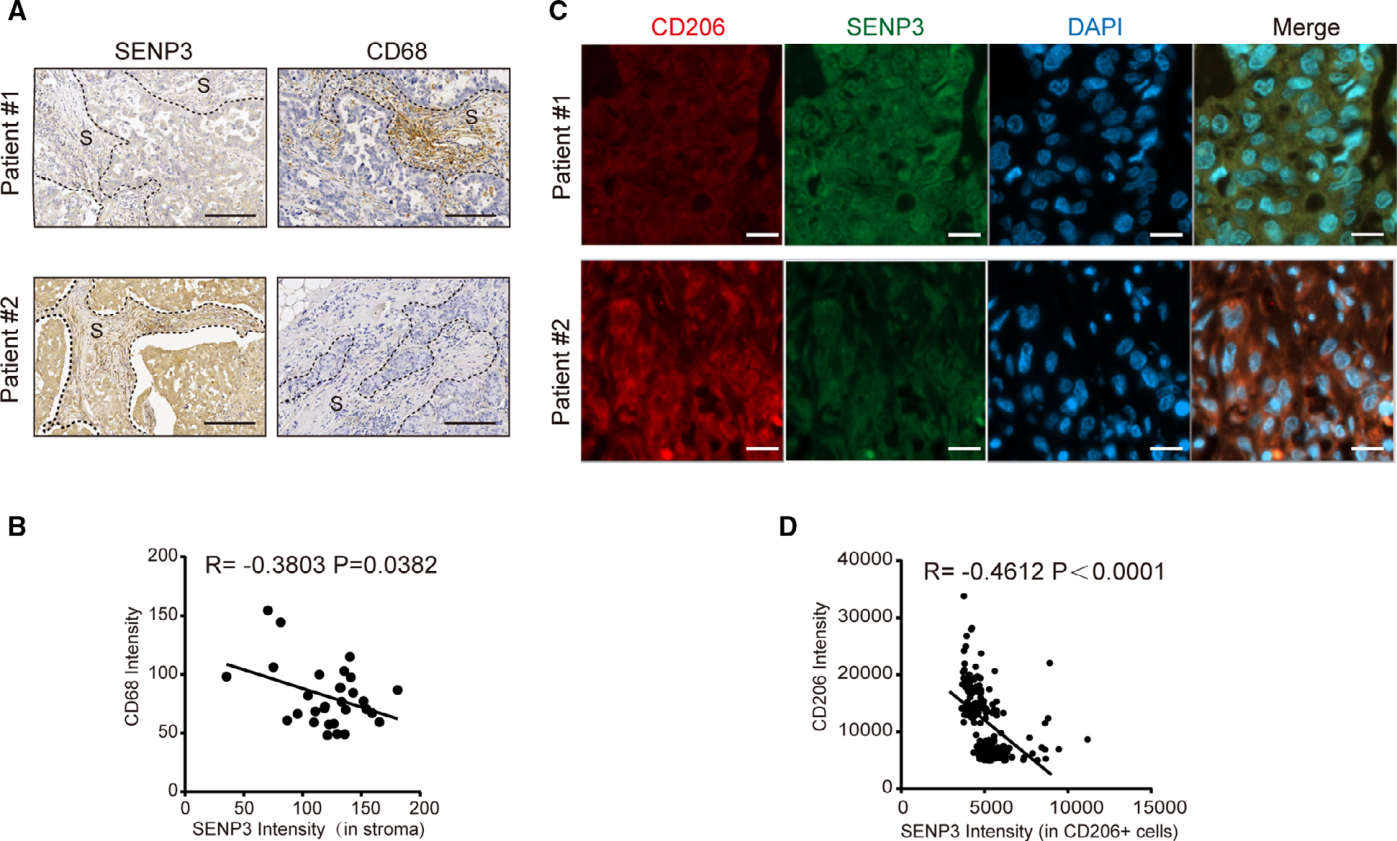
SENP3 level in macrophages is negatively correlated with M2 polarization in breast cancer patients. (A) SENP3 levels in human breast cancer tissues were detected by IHC. CD68 was used as a macrophage marker. Representative images from two patients were shown. ‘S’ denotes stroma. Scale bar: 100 μm. (B) The correlation of SENP3 intensity in tumor stroma with CD68 intensity was analyzed (*n* = 28). (C) The intensity of SENP3 and CD206 in human breast cancer tissues was determined by co‐IF. Representative images from two patients are shown. Scale bar: 50 μm. (D) The correlation of SENP3 intensity with CD206 intensity was analyzed (*n* = 245).

Given that macrophage‐specific SENP3 deletion promoted breast cancer progression in mice, we examined the level of SENP3 in macrophages in human breast cancer tissues. The IHC data showed that in the stroma area, SENP3 staining intensity was negatively correlated with the levels of CD68, a macrophage marker, with statistical significance (*n* = 28; Fig. 6A,B). Furthermore, the immunofluorescence staining validated the negative correlation between SENP3 and CD206, an M2 indicator (*n* = 245; Fig. 6C,D). Next, we evaluated the correlation between SENP3 levels in M2 macrophages and clinical outcomes, including tumor stage, clinical tumor, lymph node, metastasis (TNM) classification, and lymphatic metastasis. The statistical analysis showed that the SENP3 levels in macrophages correlated with lymphatic metastasis with a *P*‐value of 0.0051 (Table 2). A lower level of SENP3 also appeared to be associated with advanced tumor stages and TNM grades (Table 3), although the association was not statistically significant. SENP3 level showed a negative correlation (R < −0.6) with CD206 level in patients with advanced tumors (stage T3/T4, TNM grade III, and lymph node metastasis), whereas such a correlation was not seen in lower‐grade tumors (Table 4). This supports the conclusion that SENP3 downregulation in macrophage potentially accelerates the malignant progression of breast cancer.

**Table 2:** The relation between the clinicopathological features and SENP3 level in macrophages.

Characteristics	Case no.	SENP3 (average intensity in macrophages)	Parametric test value	*P*‐value
Age*				
≤ 50	141	5505.44 ± 1121.01	*t* = 2.099 *df* = 243	0.0368
>50	104	4499.58 ± 697.82		
Nodal status*				
N0	215	5144.11 ± 1126.24	*t* = 2.567 *df* = 243	0.0109
N1–2	30	4608 ± 513.17		
Clinical stage (TNM classification)				
Ⅰ	26	5549.45 ± 1659.03	*F* = 2.983 *df* = 2	0.0525
Ⅱ	184	5042.19 ± 1031.93		
Ⅲ	35	4919.31 ± 687.42		
Tumor stage				
T1	28	5437.71 ± 1648.35	*F* = 1.702 *df* = 3	0.1673
T2	163	5301.17 ± 1448.7		
T3	23	5002.06 ± 951.64		
T4	31	4990.49 ± 694.12		

* *P* < 0.05.

**Table 3:** The correlation of SENP3 in macrophages and CD206 in patients.

Characteristics	SENP3 (average intensity)	CD206 (average intensity)	*R*‐value	*P*‐value
Nodal status				
N0	5144.11 ± 1126.24	10594.24 ± 5266.87	−0.4359	0.000
N1–2	4608 ± 513.17	14874.47 ± 6645.6	−0.7233	0.000
PR status				
T1	5437.71 ± 1648.35	11811.66 ± 6054.13	−0.3316	0.0848
T2	5301.17 ± 1448.7	11253.88 ± 5734.9	−0.4781	0.000
T3	5002.06 ± 951.64	11361.85 ± 4882.06	−0.7612	0.000
T4	4990.49 ± 694.12	9598.9 ± 5059.74	−0.6206	0.0002
Clinical stage (TNM classification)				
I	5549.45 ± 1659.03	11002.52 ± 5335.12	−0.2527	0.2129
II	5042.19 ± 1031.93	11325.03 ± 5764.41	−0.5098	0.000
III	4919.31 ± 687.42	10117.86 ± 5040.39	−0.655	0.000

**Table 4:** The correlation of SENP3 in macrophages and CD206 in different subtypes of breast cancer patients. Her‐2, human epidermal growth factor receptor‐2.

Characteristics	SENP3 (average intensity)	CD206 (average intensity)	*R*‐value	*P*‐value
ER status				
Negative	4981.17 ± 1143.08	12385.34 ± 5534.24	−0.5463	0.000
Positive	5199.86 ± 997.01	9537.52 ± 5335.03	−0.315	0.0008
PR status				
Negative	5071.73 ± 1122.33	11231.61 ± 5422.24	−0.4251	0.000
Positive	5101.72 ± 948.07	10727.09 ± 6283.18	−0.6082	0.000
Her‐2 status				
Negative	5028.93 ± 1121.61	11472.45 ± 5652.07	−0.4456	0.000
Positive	5593.7 ± 1026.97	6401.79 ± 1617.84	0.0205	0.8205
Triple negative status				
ER/PR^−^Her‐2^−^	4965.3 ± 1244.99	12725.45 ± 5633.95	−0.529	0.000
ER/PR^+^Her‐2^−^	5088.39 ± 994.88	10301.42 ± 5438.1	−0.3474	0.0002
ER/PR^−^Her‐2^+^	5258.97 ± 802.6	10172.84 ± 5580	−0.6411	0.0004
ER/PR^+^Her‐2^+^	5541.84 ± 823.3	7058.83 ± 2910.75	−0.03276	0.9195

We then examined the correlation of SENP3 and CD206 among different intrinsic or molecular subtypes of breast cancer. We found a strong negative correlation between both factors in luminal A type [estrogen receptor (ER)^+^, progesterone receptor (PR)^+^, human epidermal growth factor receptor‐2 (HER2)^–^], HER2 enriched type (ER/PR^−^, HER2^+^), and in triple negative breast cancer type (TNBC), demonstrating that SENP3 could suppress M2 polarization in general (Table 3).

In addition, the TIMER analysis of breast cancer patient data revealed a weak negative correlation between the SENP3 level in the whole tumor tissues and the number of macrophages that infiltrated the tumors (Fig. S8A). Kaplan–Meier analysis showed that a high SENP3 level in breast cancer tissues was correlated with a better overall survival (Fig. S8B). Because the level of SENP3 in macrophages appeared to be comparable to that in tumor cells (Fig. 6A), these results collectively suggest that SENP3 level in macrophages, as well as in the whole breast cancer tissues, could serve as a biomarker for breast cancer progression and prognosis of breast cancer patients.

## 4 Discussion

In the present study, we uncovered a previous unnoted role of SENP3 in fine‐tuning macrophage polarization in breast cancer. Mechanistically, in SENP3‐deficient macrophages, Akt1 becomes hyper‐SUMOylated, followed by its hyper‐phosphorylation and activation, which contributes to M2 polarization in the tumor environment.

We utilized Py8119 cells derived from MMTV‐PyMT transgenic mice to generate allografts in *Senp3* cKO mice via orthotopic, subcutaneous or tail vein injection, which mimicked rapid progression of breast cancer. MMTV‐PyMT transgenic mice are a spontaneous breast cancer animal model which exhibits many features of the TNBC, such as the identity of macrophages as the major component of tumor microenvironment that contributes tumor progression [14, 34, 35]. TAM is largely believed to be correlated with poor prognosis in breast cancer, and its density within tumor lesions has also been suggested to be an independent predictor of poor outcome in various types of tumor [36, 37, 38]. Herein we discovered that the activity and polarization of TAM regulated by SENP3 facilitates tumor progression in the murine breast cancer model with Py8119. Furthermore, SENP3 abundance in stroma was negatively associated with macrophage mass in breast cancer biopsies, and SENP3 level in macrophage determined the extent of M2 polarization, eventually associated with lymphatic metastasis. Taken together, SENP3 in macrophages might be an important regulator in TNBC or more advanced tumor.

SUMOylation and SENP‐mediated de‐SUMOylation have been linked to suppressed inflammation in macrophages, exemplified by SUMOylation of PPAR γ, DICER ribonuclease III, MKK7, and NEMO/inhibitor of nuclear factor kappa B kinase regulatory subunit gamma (IKBKG) [26, 32, 41, 42]. Similarly, in our previous study we reported that SENP3 enhances expression of inflammatory cytokines in response to LPS stimulation via MKK7 de‐SUMOylation [43]. Instead, de‐SUMOylation by SENP6 had been found to suppress TLR inflammatory signaling in macrophages [25]. Therefore, dynamic SUMOylation had a complex influence on M1 polarization. However, except that the effect of SUMOylation of KLF4 on promotion of M2 polarization is limited in RAW264.7 cells, the regulatory role of protein SUMOylation or de‐SUMOylation has been less studied during the reprogramming process of macrophage polarity towards ‘pro‐tumor’ M2 subtype [9, 39, 40, 44]. Our current study has provided another example that SUMOylation facilitates M2 polarization. These findings together shed light on the roles of SUMOylation and SENPs in macrophage polarization and pro‐inflammatory or pro‐tumor responses in different contexts.

Although SENP3 was explored as a regulator of macrophage polarization in our study, how SENP3 responds to the cytokines in the tumor microenvironment still remains unclear. SENP3 has been viewed as a stress sensor, given degradation inhibition and rapid accumulation resulting from the oxidative modification upon secondary oxidative stress in multiple normal and tumor cell lines [45]. In contrast, here we observed a rapid reduction in SENP3 expression in macrophages upon the treatment with M2‐inducing cytokines IL‐4 and IL‐13.

SENP3 localization in TAM is also of interest. SENP3 is mainly targeted in cytosol, differing from its dominant localization in the nucleus in cancer cells, indicating its versatile roles in regulation of cell behavior through various targets in diverse compartments including cytosolic MKK7, Beclin 1, and dynamin‐related protein 1, as previous reported [28, 46, 47]. Consistent with its prominent cytosol distribution, SENP3 was found to control TAM polarization by targeting the cytosolic substrate Akt1. The mechanism underlying SENP3 downregulation and localization or translocalization upon M2 induction in breast cancer mouse models and patients warrants investigation in the future.

## 5 Conclusions

In summary, our study showed that SENP3 plays an important role in fine‐tuning macrophage polarization towards M2 in breast cancer via de‐SUMOylating Akt1. Furthermore, in breast cancer patients, the decrease in SENP3 in macrophages is significantly correlated with M2 polarization, lymphatic metastasis or a worse prognosis.

## Conflict of interest

The authors declare no conflicts of interest.

## Author contributions

JYi, XQS, XXS, and JYa contributed to the concept, design, and supervision. MX, XQS, XXS, and JYa wrote and reviewed the manuscript. YL, JYi, XQS, XXS, and JYa contributed to funding acquisition. All authors performed data acquisition, analysis, and interpretation, and read and approved the final manuscript. QB contributed to the design of the experiment, the development of the experiment, and the collection of data.

### Peer Review

The peer review history for this article is available at https://publons.com/publon/10.1002/1878‐0261.12967.

## Supporting information


**Fig. S1**. SENP3 deletion in macrophages promoted breast cancer progression and metastasis in the subcutaneous inoculation model with Py8119.
**Fig. S2**. SENP3 depletion had no effects on the proportion of MDSC, CD4^+^, and CD8^+^ T cells in the transplanted tumor tissues.
**Fig. S3**. SENP3 deletion in macrophages promoted melanoma progression in mouse model.
**Fig. S4**. The expression of SENP3 decreased upon IL‐4 and IL‐13 treatment in BMDM.
**Fig. S5**. The effects of SENPs on the phosphorylation of STAT6, STAT3, and Akt1.
**Fig. S6**. p‐Akt1 inhibitor effectively rescued tumor progression.
**Fig. S7**. SENP3 localized in the cytoplasm in macrophages within mouse breast cancer tissue.
**Fig. S8**. The expression of SENP3 in breast cancer was negatively related to macrophage immersion and survival rate.

## Data Availability

The data that support the findings of this study are available in the Supporting Information material of this article.

## References

[1] Binnewies M , Roberts EW , Kersten K , Chan V , Fearon DF , Merad M , Coussens LM , Gabrilovich DI , Ostrand‐Rosenberg S , Hedrick CC *et al*. (2018) Understanding the tumor immune microenvironment (TIME) for effective therapy. Nat Med 24, 541–550. 29686425 10.1038/s41591-018-0014-x PMC5998822

[2] Choi J , Cha YJ & Koo JS (2018) Adipocyte biology in breast cancer: From silent bystander to active facilitator. Prog Lipid Res 69, 11–20. 29175445 10.1016/j.plipres.2017.11.002

[3] Olson B , Li Y , Lin Y , Liu ET & Patnaik A (2018) Mouse models for cancer immunotherapy research. Cancer Discov 8, 1358–1365. 30309862 10.1158/2159-8290.CD-18-0044 PMC8725605

[4] Zhang W , Xu J , Fang H , Tang L , Chen W , Sun Q , Zhang Q , Yang F , Sun Z , Cao L *et al*. (2018) Endothelial cells promote triple‐negative breast cancer cell metastasis via PAI‐1 and CCL5 signaling. FASEB J 32, 276–288. 28899878 10.1096/fj.201700237RR

[5] Bray F , Ferlay J , Soerjomataram I , Siegel RL , Torre LA & Jemal A (2018) Global cancer statistics 2018: GLOBOCAN estimates of incidence and mortality worldwide for 36 cancers in 185 countries. CA Cancer J Clin 68, 394–424. 30207593 10.3322/caac.21492

[6] Harbeck N & Gnant M (2017) Breast cancer. Lancet 389, 1134–1150. 27865536 10.1016/S0140-6736(16)31891-8

[7] Azizi E , Carr AJ , Plitas G , Cornish AE , Konopacki C , Prabhakaran S , Nainys J , Wu K , Kiseliovas V , Setty M *et al*. (2018) Single‐cell map of diverse immune phenotypes in the breast tumor microenvironment. Cell 174, 1293–1308.e1236. 29961579 10.1016/j.cell.2018.05.060 PMC6348010

[8] Wagner J , Rapsomaniki MA , Chevrier S , Anzeneder T , Langwieder C , Dykgers A , Rees M , Ramaswamy A , Muenst S , Soysal SD *et al*. (2019) A single‐cell atlas of the tumor and immune ecosystem of human breast cancer. Cell 177, 1330–1345.e1318. 30982598 10.1016/j.cell.2019.03.005 PMC6526772

[9] Mantovani A , Sica A , Sozzani S , Allavena P , Vecchi A & Locati M (2004) The chemokine system in diverse forms of macrophage activation and polarization. Trends Immunol 25, 677–686. 15530839 10.1016/j.it.2004.09.015

[10] Murray PJ (2017) Macrophage polarization. Annu Rev Physiol 79, 541–566. 27813830 10.1146/annurev-physiol-022516-034339

[11] Saleh B , Dhaliwal HK , Portillo‐Lara R , Shirzaei Sani E , Abdi R , Amiji MM & Annabi N (2019) Local immunomodulation using an adhesive hydrogel loaded with miRNA‐laden nanoparticles promotes wound healing. Small 15, e1902232. 31328877 10.1002/smll.201902232 PMC6726510

[12] Cao M , Yan H , Han X , Weng L , Wei Q , Sun X , Lu W , Wei Q , Ye J , Cai X *et al*. (2019) Ginseng‐derived nanoparticles alter macrophage polarization to inhibit melanoma growth. J Immunother Cancer 7, 326. 31775862 10.1186/s40425-019-0817-4 PMC6882204

[13] Lin EY & Pollard JW (2007) Tumor‐associated macrophages press the angiogenic switch in breast cancer. Cancer Res 67, 5064–5066. 17545580 10.1158/0008-5472.CAN-07-0912

[14] Lin EY , Li JF , Gnatovskiy L , Deng Y , Zhu L , Grzesik DA , Qian H , Xue XN & Pollard JW (2006) Macrophages regulate the angiogenic switch in a mouse model of breast cancer. Cancer Res 66, 11238–11246. 17114237 10.1158/0008-5472.CAN-06-1278

[15] Vuong L , Kouverianou E , Rooney CM , McHugh BJ , Howie SEM , Gregory CD , Forbes SJ , Henderson NC , Zetterberg FR , Nilsson UJ *et al*. (2019) An orally active galectin‐3 antagonist inhibits lung adenocarcinoma growth and augments response to PD‐L1 blockade. Cancer Res 79, 1480–1492. 30674531 10.1158/0008-5472.CAN-18-2244

[16] Han ZJ , Feng YH , Gu BH , Li YM & Chen H (2018) The post‐translational modification, SUMOylation, and cancer (Review). Int J Oncol 52, 1081–1094. 29484374 10.3892/ijo.2018.4280 PMC5843405

[17] Zucchelli C , Tamburri S , Filosa G , Ghitti M , Quilici G , Bachi A & Musco G (2019) Sp140 is a multi‐SUMO‐1 target and its PHD finger promotes SUMOylation of the adjacent Bromodomain. Biochim Biophys Acta Gen Subj 1863, 456–465. 30465816 10.1016/j.bbagen.2018.11.011

[18] Drag M & Salvesen GS (2008) DeSUMOylating enzymes–SENPs. IUBMB Life 60, 734–742. 18666185 10.1002/iub.113

[19] Yeh ET (2009) SUMOylation and De‐SUMOylation: wrestling with life’s processes. J Biol Chem 284, 8223–8227. 19008217 10.1074/jbc.R800050200 PMC2659178

[20] Gareau JR & Lima CD (2010) The SUMO pathway: emerging mechanisms that shape specificity, conjugation and recognition. Nat Rev Mol Cell Biol 11, 861–871. 21102611 10.1038/nrm3011 PMC3079294

[21] Geiss‐Friedlander R & Melchior F (2007) Concepts in sumoylation: a decade on. Nat Rev Mol Cell Biol 8, 947–956. 18000527 10.1038/nrm2293

[22] Decque A , Joffre O , Magalhaes JG , Cossec JC , Blecher‐Gonen R , Lapaquette P , Silvin A , Manel N , Joubert PE , Seeler JS *et al*. (2016) Sumoylation coordinates the repression of inflammatory and anti‐viral gene‐expression programs during innate sensing. Nat Immunol 17, 140–149. 26657003 10.1038/ni.3342

[23] Tomasi ML , Ramani K & Ryoo M (2016) Ubiquitin‐conjugating enzyme 9 phosphorylation as a novel mechanism for potentiation of the inflammatory response. Am J Pathol 186, 2326–2336. 27561301 10.1016/j.ajpath.2016.05.007 PMC5012469

[24] Begitt A , Droescher M , Knobeloch KP & Vinkemeier U (2011) SUMO conjugation of STAT1 protects cells from hyperresponsiveness to IFNγ. Blood 118, 1002–1007. 21636857 10.1182/blood-2011-04-347930

[25] Liu X , Chen W , Wang Q , Li L & Wang C (2013) Negative regulation of TLR inflammatory signaling by the SUMO‐deconjugating enzyme SENP6. PLoS Pathog 9, e1003480. 23825957 10.1371/journal.ppat.1003480 PMC3694847

[26] Pascual G , Fong AL , Ogawa S , Gamliel A , Li AC , Perissi V , Rose DW , Willson TM , Rosenfeld MG & Glass CK (2005) A SUMOylation‐dependent pathway mediates transrepression of inflammatory response genes by PPAR‐gamma. Nature 437, 759–763. 16127449 10.1038/nature03988 PMC1464798

[27] Tillmanns S , Otto C , Jaffray E , Du Roure C , Bakri Y , Vanhille L , Sarrazin S , Hay RT & Sieweke MH (2007) SUMO modification regulates MafB‐driven macrophage differentiation by enabling Myb‐dependent transcriptional repression. Mol Cell Biol 27, 5554–5564. 17548468 10.1128/MCB.01811-06 PMC1952098

[28] Lao Y , Yang K , Wang Z , Sun X , Zou Q , Yu X , Cheng J , Tong X , Yeh ETH , Yang J *et al*. (2018) DeSUMOylation of MKK7 kinase by the SUMO2/3 protease SENP3 potentiates lipopolysaccharide‐induced inflammatory signaling in macrophages. J Biol Chem 293, 3965–3980. 29352108 10.1074/jbc.M117.816769 PMC5857993

[29] Yan S , Sun X , Xiang B , Cang H , Kang X , Chen Y , Li H , Shi G , Yeh ET , Wang B *et al*. (2010) Redox regulation of the stability of the SUMO protease SENP3 via interactions with CHIP and Hsp90. EMBO J 29, 3773–3786. 20924358 10.1038/emboj.2010.245 PMC2989103

[30] Gibby K , You WK , Kadoya K , Helgadottir H , Young LJ , Ellies LG , Chang Y , Cardiff RD & Stallcup WB (2012) Early vascular deficits are correlated with delayed mammary tumorigenesis in the MMTV‐PyMT transgenic mouse following genetic ablation of the NG2 proteoglycan. Breast Cancer Res 14, R67. 22531600 10.1186/bcr3174 PMC3446402

[31] Zhang Y , Yang K , Yang J , Lao Y , Deng L , Deng G , Yi J , Sun X & Wang Q (2020) SENP3 suppresses osteoclastogenesis by de‐conjugating SUMO2/3 from IRF8 in bone marrow‐derived monocytes. Cell Rep 30, 1951–1963.e1954. 32049023 10.1016/j.celrep.2020.01.036

[32] Chang TH , Xu S , Tailor P , Kanno T & Ozato K (2012) The small ubiquitin‐like modifier‐deconjugating enzyme sentrin‐specific peptidase 1 switches IFN regulatory factor 8 from a repressor to an activator during macrophage activation. J Immunol 189, 3548–3556. 22942423 10.4049/jimmunol.1201104 PMC4158928

[33] Li R , Wei J , Jiang C , Liu D , Deng L , Zhang K & Wang P (2013) Akt SUMOylation regulates cell proliferation and tumorigenesis. Cancer Res 73, 5742–5753. 23884910 10.1158/0008-5472.CAN-13-0538

[34] Lin EY , Nguyen AV , Russell RG & Pollard JW (2001) Colony‐stimulating factor 1 promotes progression of mammary tumors to malignancy. J Exp Med 193, 727–740. 11257139 10.1084/jem.193.6.727 PMC2193412

[35] Wyckoff J , Wang W , Lin EY , Wang Y , Pixley F , Stanley ER , Graf T , Pollard JW , Segall J & Condeelis J (2004) A paracrine loop between tumor cells and macrophages is required for tumor cell migration in mammary tumors. Cancer Res 64, 7022–7029. 15466195 10.1158/0008-5472.CAN-04-1449

[36] Leek RD & Harris AL (2002) Tumor‐associated macrophages in breast cancer. J Mammary Gland Biol Neoplasia 7, 177–189. 12463738 10.1023/a:1020304003704

[37] Dave SS , Wright G , Tan B , Rosenwald A , Gascoyne RD , Chan WC , Fisher RI , Braziel RM , Rimsza LM , Grogan TM *et al*. (2004) Prediction of survival in follicular lymphoma based on molecular features of tumor‐infiltrating immune cells. N Engl J Med 351, 2159–2169. 15548776 10.1056/NEJMoa041869

[38] Farinha P , Masoudi H , Skinnider BF , Shumansky K , Spinelli JJ , Gill K , Klasa R , Voss N , Connors JM & Gascoyne RD (2005) Analysis of multiple biomarkers shows that lymphoma‐associated macrophage (LAM) content is an independent predictor of survival in follicular lymphoma (FL). Blood 106, 2169–2174. 15933054 10.1182/blood-2005-04-1565

[39] Ruffell B & Coussens LM (2015) Macrophages and therapeutic resistance in cancer. Cancer Cell 27, 462–472. 25858805 10.1016/j.ccell.2015.02.015 PMC4400235

[40] Zhou D , Huang C , Lin Z , Zhan S , Kong L , Fang C & Li J (2014) Macrophage polarization and function with emphasis on the evolving roles of coordinated regulation of cellular signaling pathways. Cell Signal 26, 192–197. 24219909 10.1016/j.cellsig.2013.11.004

[41] Gross TJ , Powers LS , Boudreau RL , Brink B , Reisetter A , Goel K , Gerke AK , Hassan IH & Monick MM (2014) A microRNA processing defect in smokers’ macrophages is linked to SUMOylation of the endonuclease DICER. J Biol Chem 289, 12823–12834. 24668803 10.1074/jbc.M114.565473 PMC4007470

[42] Im SS & Osborne TF (2011) Liver X receptors in atherosclerosis and inflammation. Circ Res 108, 996–1001. 21493922 10.1161/CIRCRESAHA.110.226878 PMC3082200

[43] Chen X , Lao Y , Yi J , Yang J , He S & Chen Y (2020) SENP3 in monocytes/macrophages up‐regulates tissue factor and mediates lipopolysaccharide‐induced acute lung injury by enhancing JNK phosphorylation. J Cell Mol Med 24, 5454–5462. 32237051 10.1111/jcmm.15199 PMC7214145

[44] Wang K , Zhou W , Cai Q , Cheng J , Cai R & Xing R (2017) SUMOylation of KLF4 promotes IL‐4 induced macrophage M2 polarization. Cell Cycle 16, 374–381. 28059602 10.1080/15384101.2016.1269045 PMC5324751

[45] Huang C , Han Y , Wang Y , Sun X , Yan S , Yeh ET , Chen Y , Cang H , Li H , Shi G *et al*. (2009) SENP3 is responsible for HIF‐1 transactivation under mild oxidative stress via p300 de‐SUMOylation. EMBO J 28, 2748–2762. 19680224 10.1038/emboj.2009.210 PMC2750016

[46] Anderson CA & Blackstone C (2013) SUMO wrestling with Drp1 at mitochondria. EMBO J 32, 1496–1498. 23632859 10.1038/emboj.2013.103 PMC3671251

[47] Liu K , Guo C , Lao Y , Yang J , Chen F , Zhao Y , Yang Y , Yang J & Yi J (2020) A fine‐tuning mechanism underlying self‐control for autophagy: deSUMOylation of BECN1 by SENP3. Autophagy 16, 975–990. 31373534 10.1080/15548627.2019.1647944 PMC7469448

