# Supplementary material for: SENP3 loss promotes M2 macrophage polarization and breast cancer progression
Source: Mol Oncol. 2021 May 29;16(4):1026–44. doi: 10.1002/1878-0261.12967 (PMC8847990; doi:10.1002/1878-0261.12967)
Supplement: Supplementary file 1 — Fig. S1. SENP3 deletion in macrophages promoted breast cancer progression and metastasis in the subcutaneous inoculation model with Py8119. Fig. S2. SENP3 depletion had no effects on the proportion of MDSC, CD4+, and CD8+ T cells in the transplanted tumor tissues. Fig. S3. SENP3 deletion in macrophages promoted melanoma progression in mouse model. Fig. S4. The expression of SENP3 decreased upon IL‐4 and IL‐13 treatment in BMDM. Fig. S5. The effects of SENPs on the phosphorylation of STAT6, STAT3, and Akt1. Fig. S6. p‐Akt1 inhibitor effectively rescued tumor progression. Fig. S7. SENP3 localized in the cytoplasm in macrophages within mouse breast cancer tissue. Fig. S8. The expression of SENP3 in breast cancer was negatively related to macrophage immersion and survival rate. [file MOL2-16-1026-s001.pdf]

Supplemental Figure1

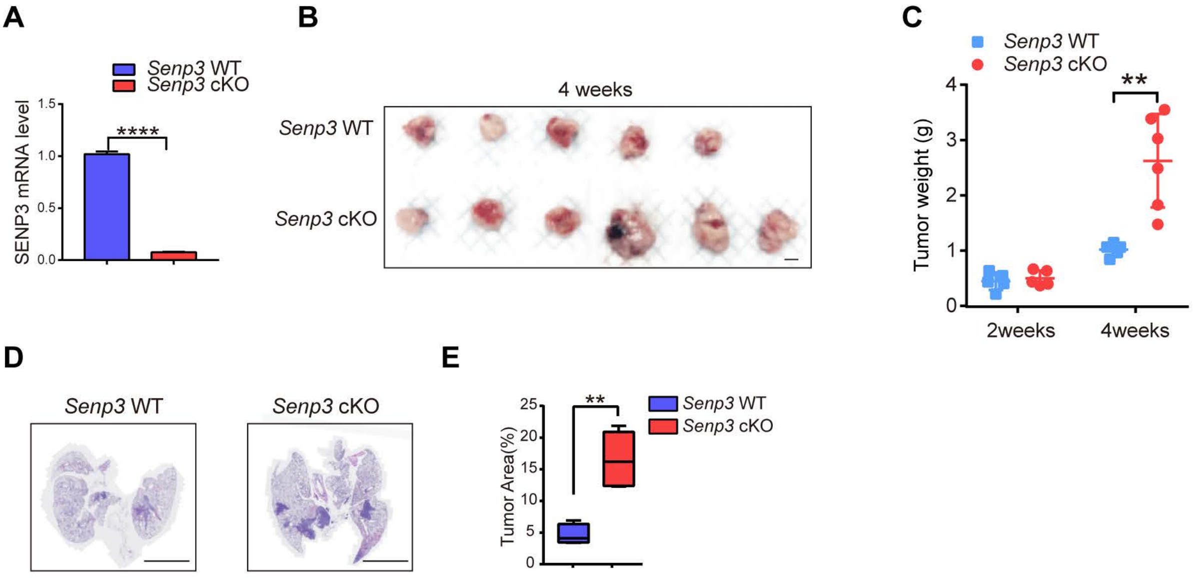

Supplemental Figure2

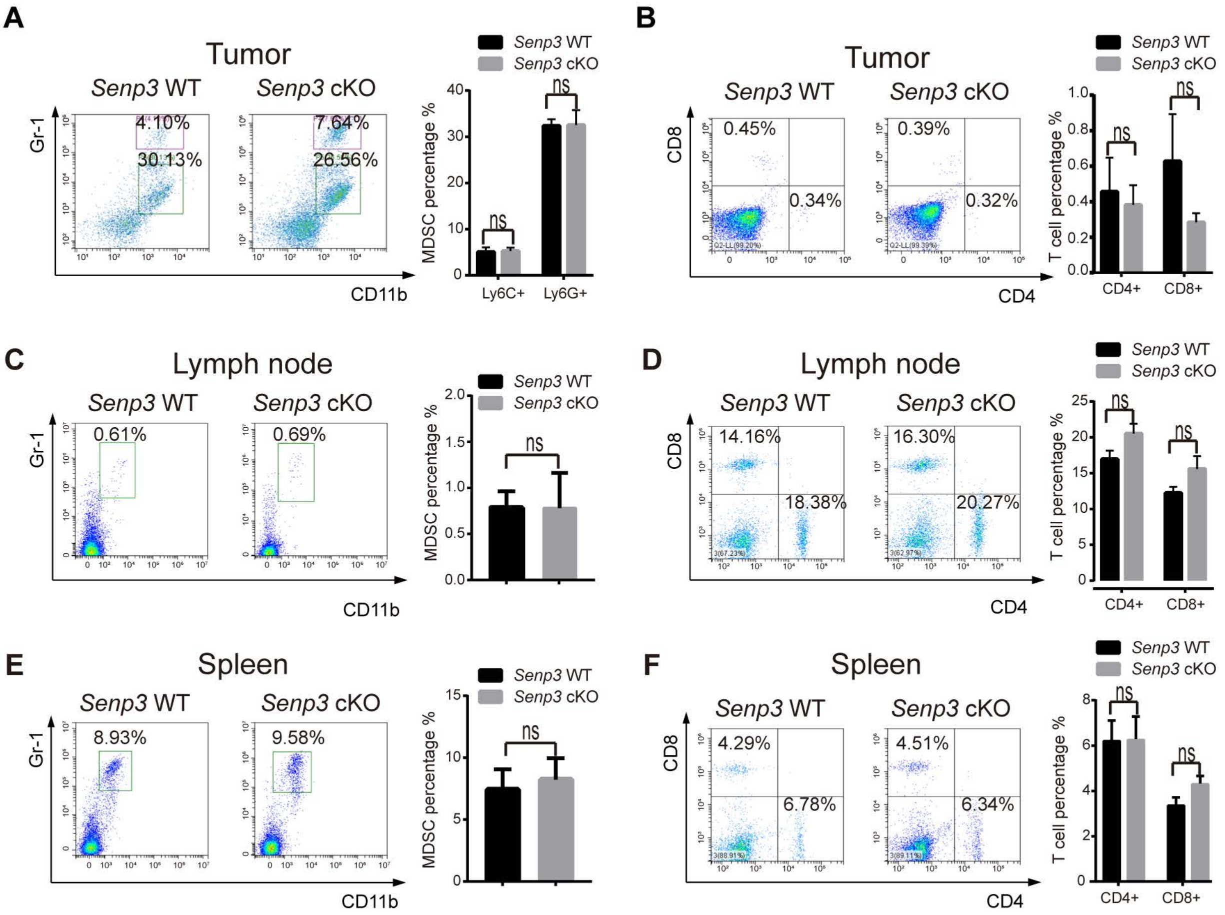

Supplemental Figure 3

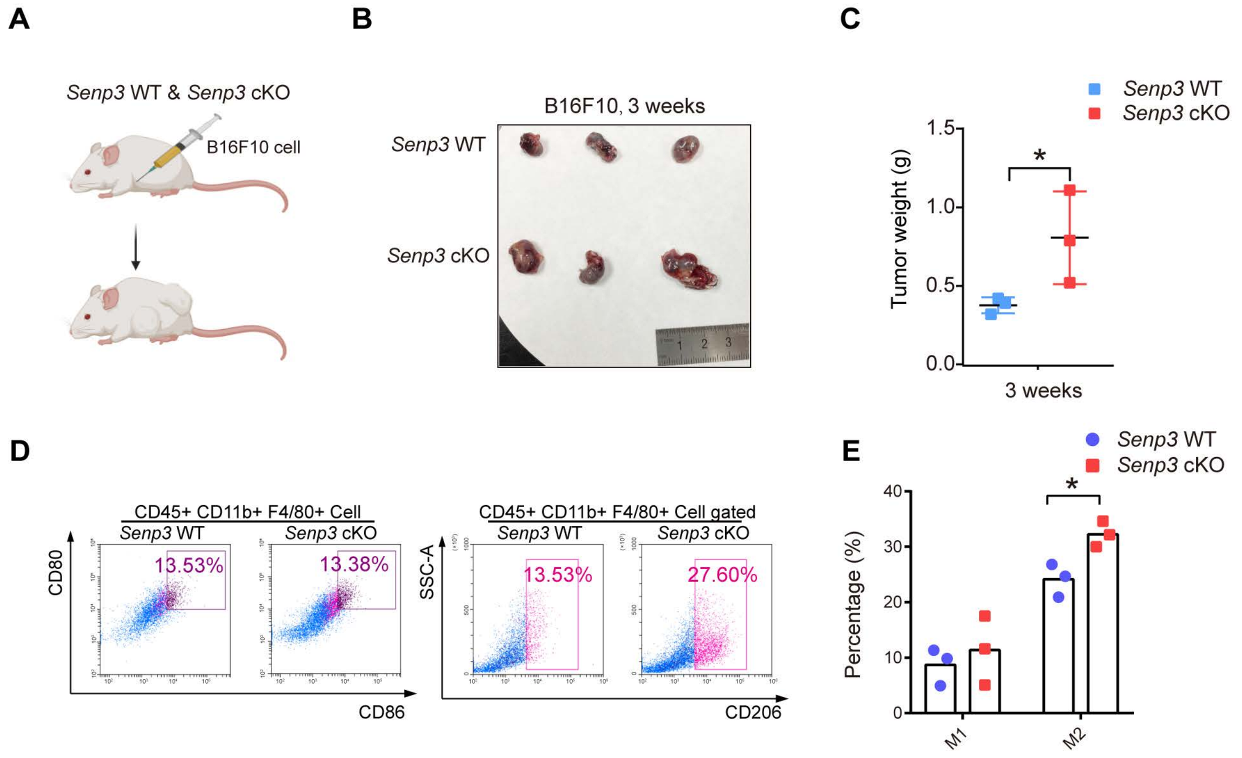

Supplemental Figure 4

A

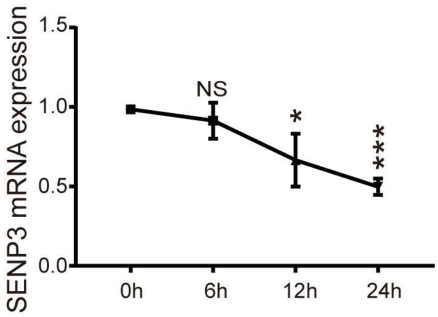

Supplemental Figure 5

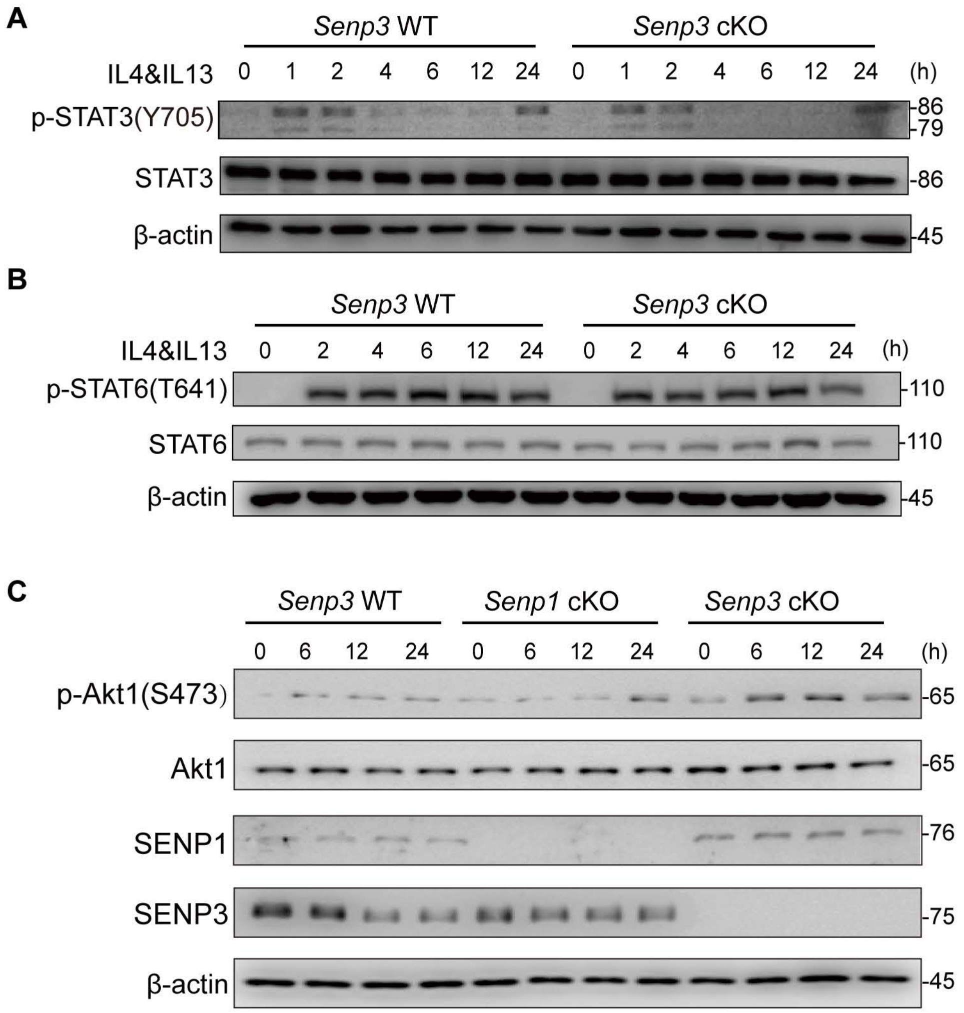

Supplemental Figure 6

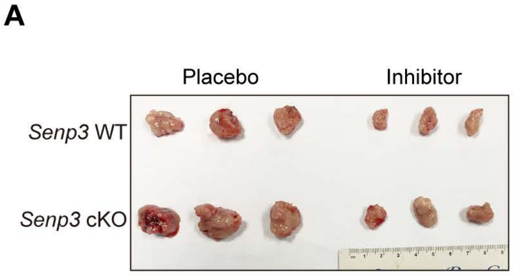

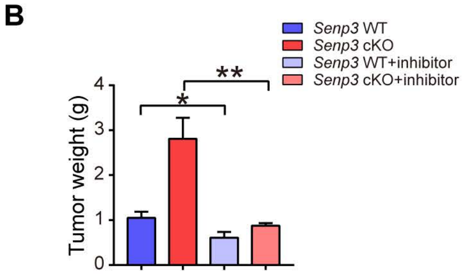

Supplemental Figure 7

A

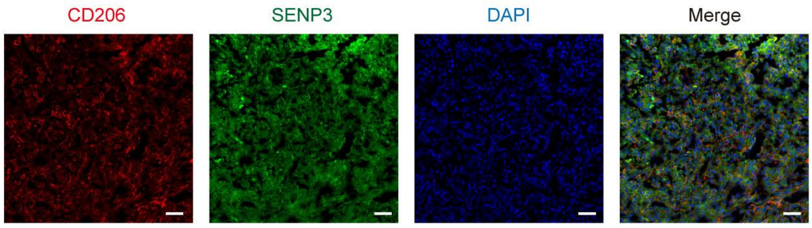

Supplemental Figure 8

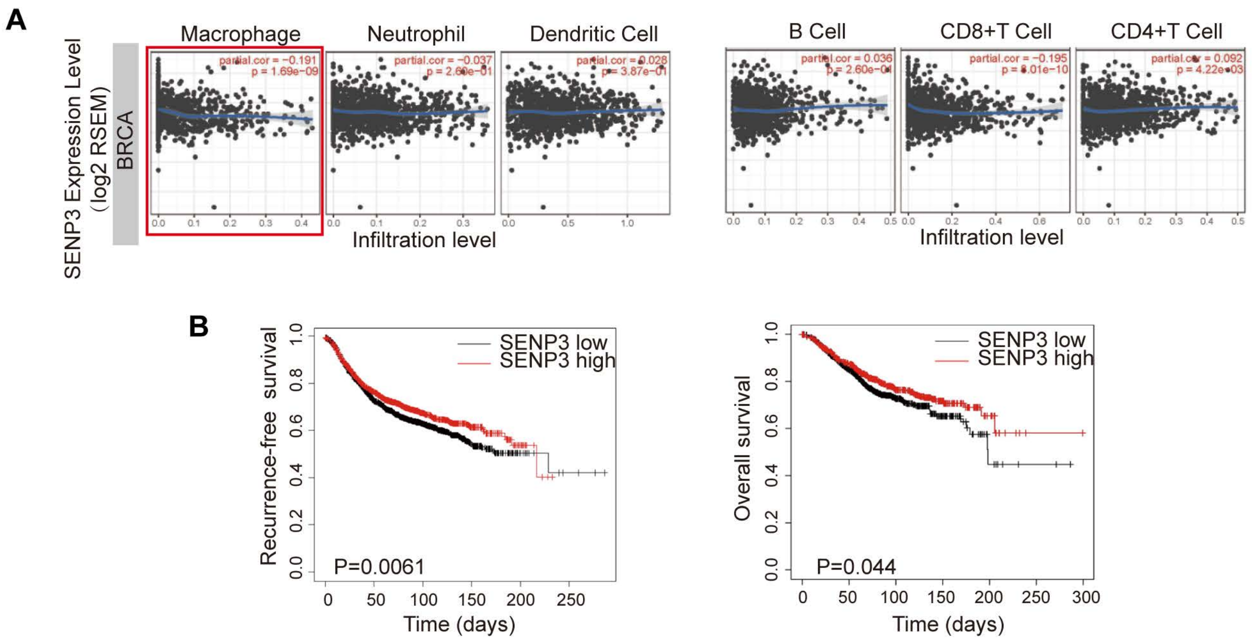

SENP3 is a regulator of macrophage polarization for breast cancer progression

Xiao et al.

Supporting Information

Supplemental figure legends

Supplemental Figure 1. SENP3 deletion in macrophages promoted breast cancer progression and metastasis in subcutaneous model.

(A) QPCR detection of knockout efficiency of *Senp3* cKO mice (**** P<0.0001). (B)Transplanted tumor size at 4 weeks post inoculation. Scale bar, 1cm (C) Orthotopic transplanted tumor weight in 2 and 4 weeks. (D, E) Lung metastasis from *Senp3* WT and *Senp3* cKO mice in 4 weeks. Scale bar, 1cm. Graphs show the mean±s.d. (n=5 in *Senp3* WT, n=6 in *Senp3* cKO, **P<0.01).

Supplemental Figure 2. SENP3 depletion had no effects on the proportion of MDSC, CD4+ and CD8+ T cells in the transplanted tumor tissues.

The percentages of MDSCs (CD11b+Gr1+), CD4+ T cells and CD8+ T cells were measured by flow cytometry. The graphs were shown with mean±s.d. (n=5 in *Senp3* WT, n=6 in *Senp3* cKO). (A, B) In tumor tissues. (C, D) In spleen. (E, F) In tumor draining lymph nodes. Ns: no significance.

Supplemental Figure3. SENP3 deletion in macrophages promoted melanoma progression in mouse model.

(A)Schema for melanoma mouse model. 5×10^5 B16F10 cells were subcutaneously injected into *Senp3* WT and *Senp3* cKO mice. (B) Transplanted

tumor size at 3 weeks post inoculation, Scale bar, 1cm. (C) Transplanted tumor weight at 3 weeks (*P<0.05). (D-E)Three-week transplanted melanoma tissues were digested. Isolated cells were analyzed and gated with bone marrow derived macrophage markers CD45+ CD11b+ F4/80+ by flow cytometry. M1 macrophages with CD80+ CD86+ and M2 macrophages with CD206+ were analyzed. Quantification was performed and graphs showed the mean±s.d. (n=3 in *Senp3* WT, n= 3 in *Senp3* cKO, *P<0.05).

Supplemental Figure 4. The expression of SENP3 was decreased upon IL4&IL13 treatment in BMDMs.

BMDMs were treated with IL4&IL13 (20ng/ml) for 24 hours. The expression of SENP3 was analyzed by qRT-PCR. The experiment repeated three times (NS: no significance, *P<0.05, *** P<0.001).

Supplemental Figure 5. Macrophage SENP3 localizes to the cytoplasm in mouse tumor tissue; SENP3 depletion did not change the phosphorylation of STAT6 and STAT3; Senp1 knockout in macrophages induced by IL4&IL13 did not promote the phosphorylation of Akt1.

(A) CD206(Red) and SENP3(Green) in macrophages in transplanted tumor tissues were evaluated by co-immunofluorescence. The representative images were shown. Scale bar, 20μm. Phosphorylated STAT3 at Y705 (p-STAT3 for short) and STAT6 at T641 (p-STAT6 for short) in BMDMs were measured by Western blot. (B) p-STAT3. (C) p-STAT6. (D)SENP3, SENP1, Akt1, P-Akt1 level was measured by Western blot. (*Senp3* WT, *Senp1* cKO, *Senp3* cKO) BMDMs were treated with IL4 and IL13 for indicated times.

Supplemental Figure 6. p-Akt1 inhibitor effectively inhibits tumor progression.

(A)Transplanted tumor size at 4 weeks post inoculation. Scale bar, 1cm. (B) Subcutaneous transplanted tumor weight 4 weeks (* P<0.05, ** P<0.01).

Supplemental Figure 7. The expression of SENP3 in breast cancer was negatively related to macrophage immersion and survival period.

(A)The correlation between the expression of SENP3 and the infiltration level of different populations of immune cell in breast cancer was evaluated by TIMER analysis. (B) Recurrence-free survival and overall curve for breast cancer patients with high or low SENP3 expression. Adopted from TCGA, n=1402 and n=3955 respectively.
